# Supplementary material for: Decreased Enhancer-Promoter Proximity Accompanying Enhancer Activation
Source: Mol Cell. 2019 Nov 7;76(3):473–484.e7. doi: 10.1016/j.molcel.2019.07.038 (PMC6838673; doi:10.1016/j.molcel.2019.07.038)
Supplement: Document S1. Figures S1–S5 and Tables S1–S10 [file mmc1.pdf]

Molecular Cell, Volume *76*

Supplemental Information

Decreased Enhancer-Promoter Proximity

Accompanying Enhancer Activation

Nezha S. Benabdallah, Iain Williamson, Robert S. Illingworth, Lauren Kane, Shelagh Boyle, Dipta Sengupta, Graeme R. Grimes, Pierre Therizols, and Wendy A. Bickmore

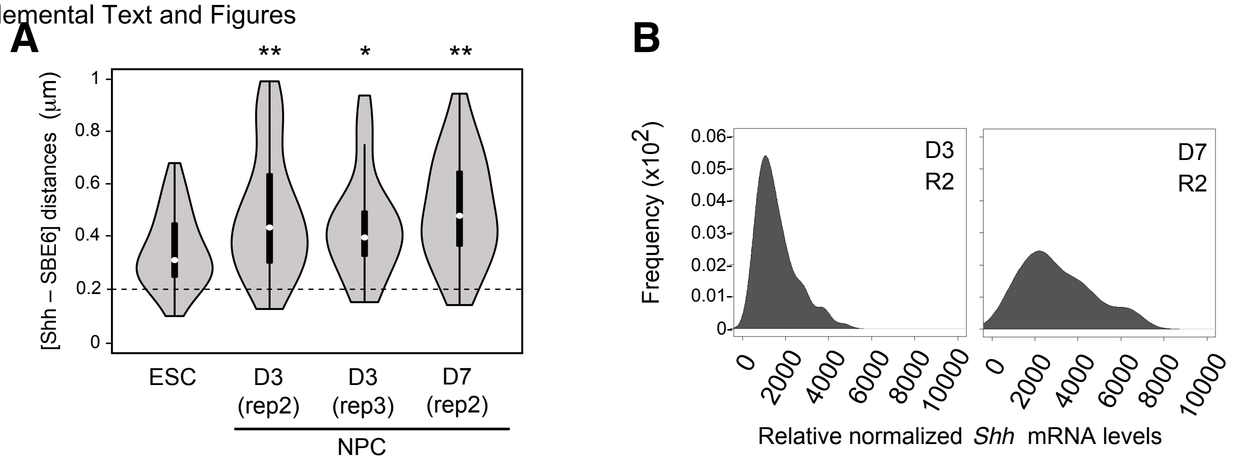

Supplementary Figure 1

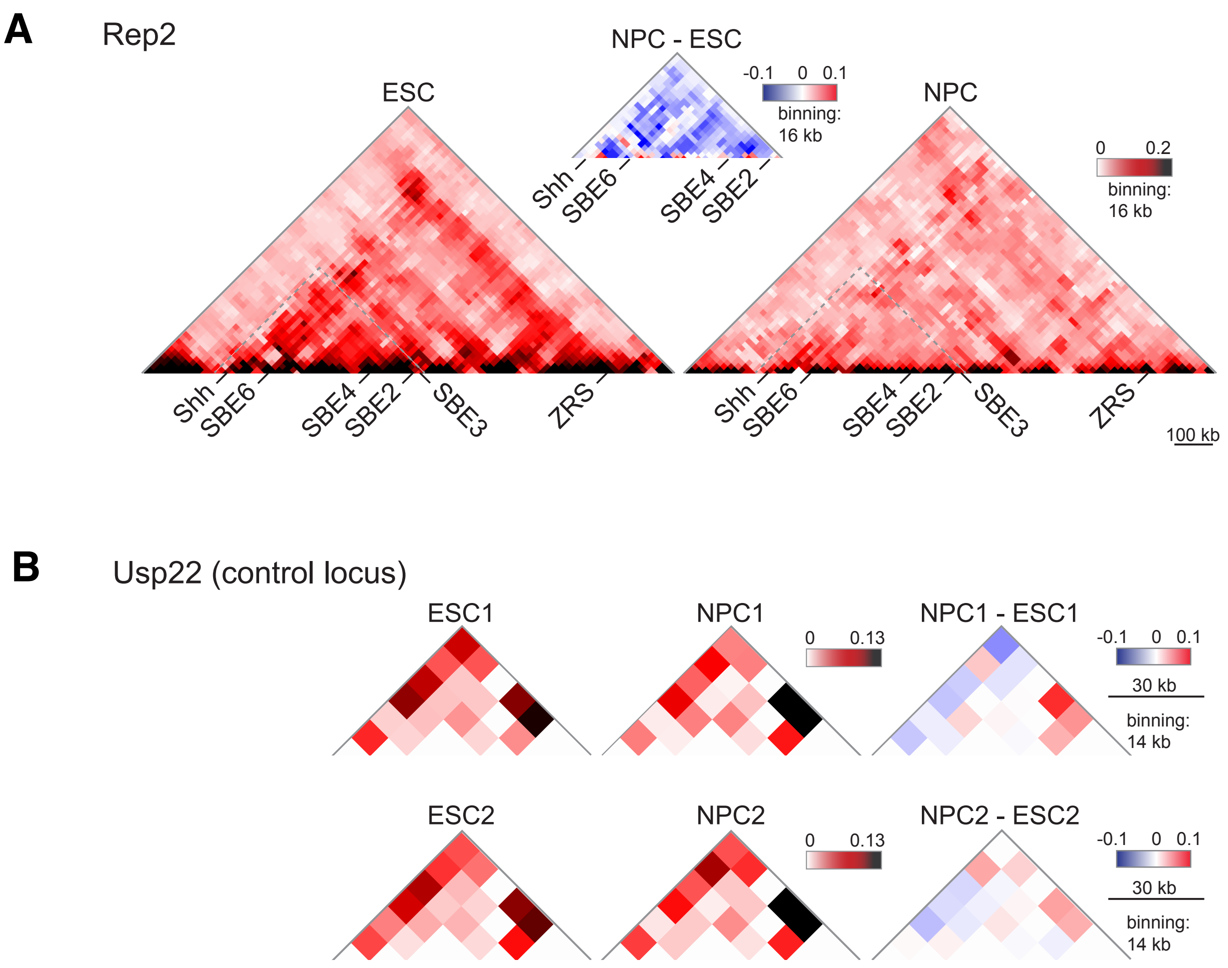
A
Rep2
NPC - ESC
-0.1 0 0.1
binning: 16 kb
ESC
NPC
0 0.2
binning: 16 kb
Shh
SBE6
SBE4
SBE2
SBE3
ZRS
100 kb
B
Usp22 (control locus)
ESC1
NPC1
0 0.13
NPC1 - ESC1
-0.1 0 0.1
30 kb
binning: 14 kb
ESC2
NPC2
0 0.13
NPC2 - ESC2
-0.1 0 0.1
30 kb
binning: 14 kb

Figure S2

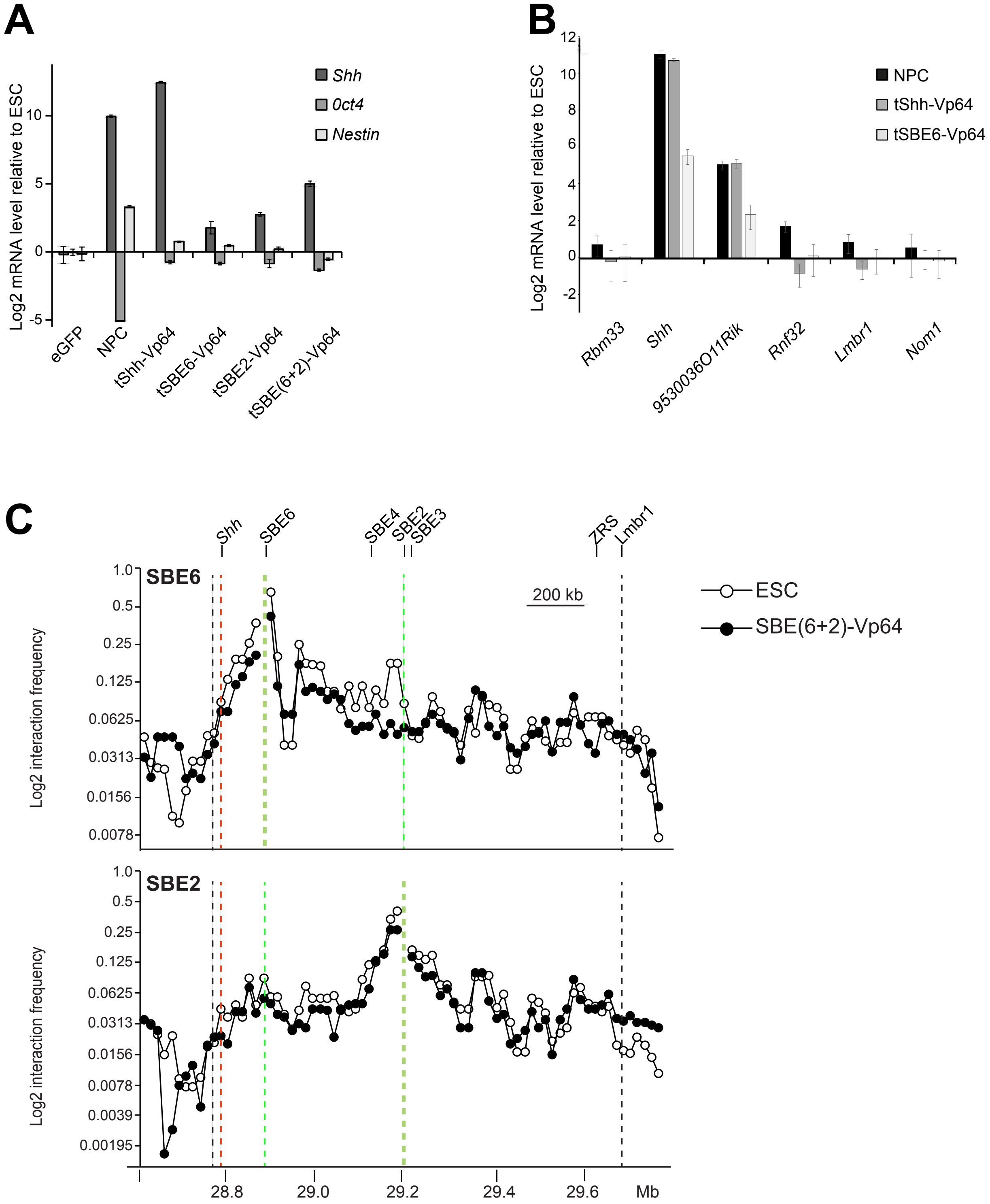
A
Log2 mRNA level relative to ESC
Shh
Oct4
Nestin
eGFP
NPC
tShh-Vp64
tSBE6-Vp64
tSBE2-Vp64
tSBE(6+2)-Vp64
B
Log2 mRNA level relative to ESC
NPC
tShh-Vp64
tSBE6-Vp64
Rbm33
Shh
9530036O11Rik
Rnf32
Lmbr1
Nom1
C
Shh
SBE6
SBE4
SBE2
SBE3
ZRS
Lmbr1
SBE6
SBE2
200 kb
ESC
SBE(6+2)-Vp64
Log2 interaction frequency
Mb

Figure S3

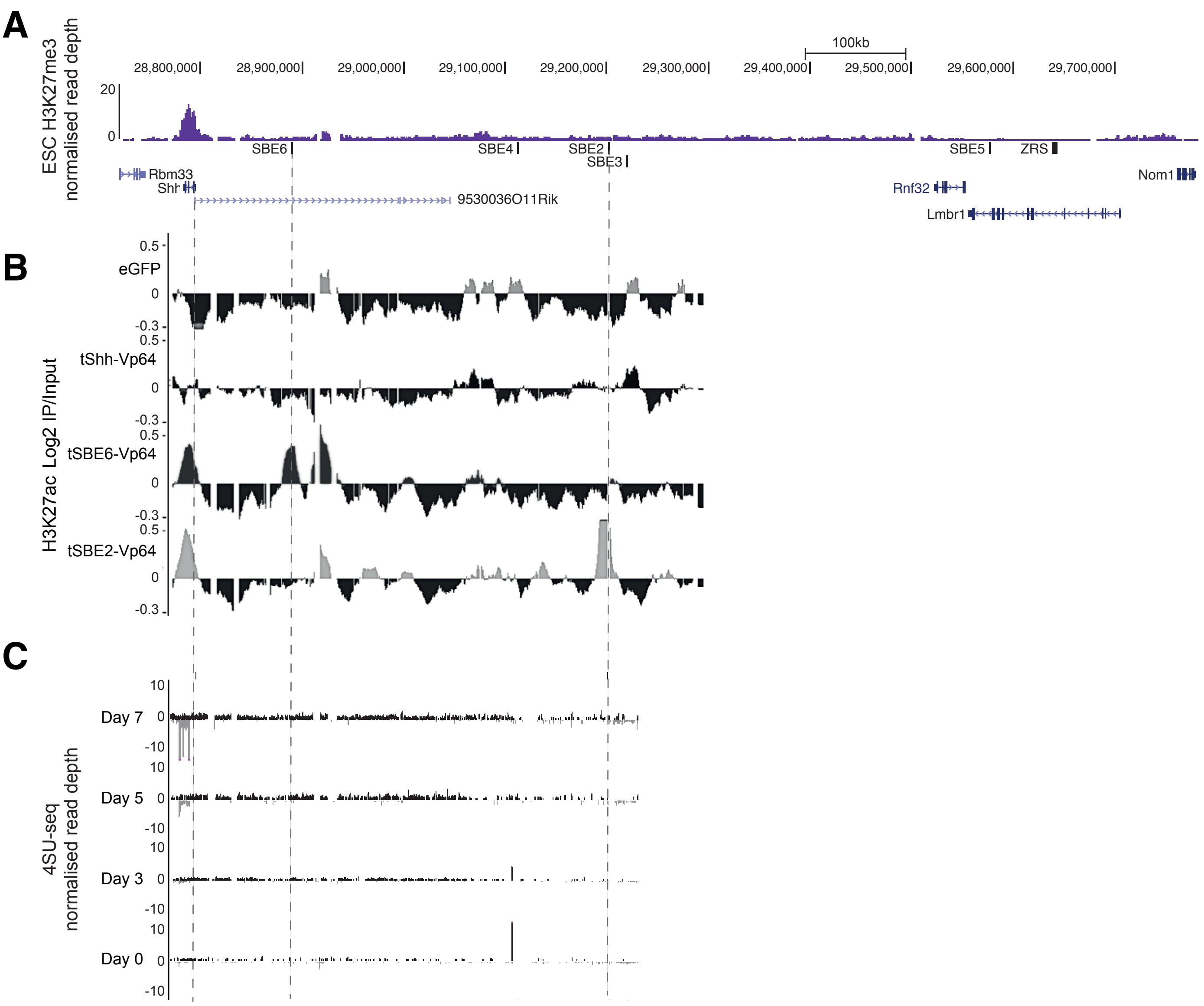

Supplementary Figure 4

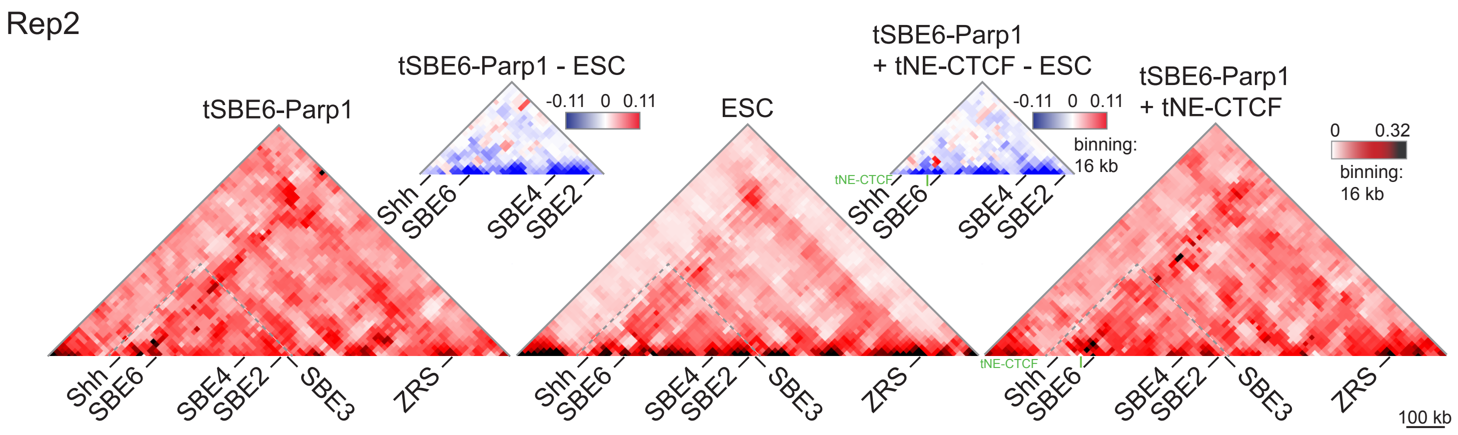
Rep2
tSBE6-Parp1
tSBE6-Parp1 - ESC
-0.11 0 0.11
ESC
tSBE6-Parp1
+ tNE-CTCF - ESC
-0.11 0 0.11
binning:
16 kb
tSBE6-Parp1
+ tNE-CTCF
0 0.32
binning:
16 kb
tNE-CTCF
Shh
SBE6
SBE4
SBE2
SBE3
ZRS
100 kb

Figure S5

Supplementary Figures

Figure S1. FISH and Shh expression during NPC differentiation. Related to Figure 1.

A) Violin plots representing Shh-SBE6 inter-probe distances after FISH in ESCs and in NPCs differentiated for 3 (D3) and 7 (D7) days of differentiation. Data are from the second (rep 2) and third (rep 3) biological replicates of the experiment shown in Figure 1. * $p < 0.05$, ** $p < 0.01$. B) Kernel density plots showing *Shh* mRNA expression in single NPCs relative to *Gapdh* and normalised to expression in ESC at D3 and D7 of differentiation for a second biological replicate. Statistical data relating to FISH data from this figure are included in Table S1.

Figure S2. Chromosome conformation capture of the Shh regulatory region. Related to Figure 2.

A) 5C heat-maps from ESCs and NPCs for the *Shh* regulatory region (mm9, chr5:28604000-29780000) with 16kb binning and smoothing (biological replicate of experiment in Figure 2). Difference 5C plots are shown above the main 5C plots. B) 5C heat maps from the control Usp22 locus.

Figure S3. Inducing Shh expression in ESCs does not perturb pluripotency. Related to Figure 3.

A) qRT-PCR showing mean (± SEM) log2 mRNA levels of *Shh*, *Oct4* and *Nestin* relative to *Gapdh* and normalized to eGFP expressing ESCs, in NPCs and in ESCs expressing tShh-Vp64, tSBE6-Vp64, tSBE2-Vp64 and tSBE(6+2)-Vp64. Data are from one biological replicate. B) As in (A) but for genes from the Shh TAD and the flanking TADs. Data in A and B relate to main Figure 3. C) Virtual 4C visualisation of 5C data from main Figure 3E, for ESCs and for ESCs expression SBE(6+2)-Vp64 with SBE6 and SBE2 viewpoints. Genome co-ordinates (Mb) are from the mm9 assembly of the mouse genome.

Figure S4. Chromatin and transcription profiling of the Shh regulatory region. Related to Figure 3.

A) Normalised read counts for H3K27me3 ChIP-seq across the *Shh* regulatory region. The position of Shh enhancers (SBE2-6 and ZRS) as well as the position of genes in the region is shown below the ChIP track. Genome co-ordinates are from the mm9 assembly of the mouse genome. NCBI GEO accession number for these data: GSE89512. Data are from Illingworth et al., 2015; GEO accession GSE69955). B) Log2 ratio H3K27ac ChIP: input of MNase digested chromatin, hybridised to an array covering the

Shh regulatory region. Mapping is aligned to the genome browser track shown in (A). Data are from; ESCs expressing control eGFP, or TALE-Vp64 fusions targeting the *Shh* promoter (tShh-Vp64), SBE6 (tSBE6-Vp64) or SBE2 (tSBE2-Vp64). Dotted lines represent location of the *Shh* promoter, SBE6 and SBE2. NCBI GEO accession number for these data: GSE89512. C) Normalised read counts for 4SU-seq during a time course of NPC differentiation from day 0 to day 7. Tracks are aligned to the genome co-ordinates in (A). NCBI GEO accession number for these data: GSE115774.

Figure S5. Chromosome conformation capture of the* Shh *regulatory region in wild type, tSBE6-PARP1 and tSBE6-PARP1 + tNE-CTCF ESCs. Related to Figure 6.

Biological replicate for experiment in Figure 6G. 5C heatmaps of the *Shh* regulatory region (mm9, chr5: 28604000-29780000) with 16kb binning and smoothing. Difference 5C plots are shown above the main 5C plots.

Table S1, Mann-Whitney U significance tests for inter-probe FISH distances across the Shh regulatory domain during NPC differentiation. Related to Figure 1

Table showing the *p*-values of each biological replicate for differences between the inter-probe distances from ESCs against the data from various days of NPC differentiation. Values in bold are significant ($p<0.05$). Also shown are the % alleles (probe pairs) where probes are considered co-localised (i.e. distances of <200nm) for data from ESCs and NPCs. 40 to 100 loci were analysed per experiment. Unless otherwise indicated, imaged were captured using structured illumination microscopy (SIM).

**Probe pairs**	**Day of NPC differentiation**	***p***	**Imaging mode**	**% of probe pair <200nm in ESCs**	**% of probe pair <200nm in NPCs**
**Shh-SBE6**	**3**	0.55	SIM	26.3	25.4
		**0.018**	SIM	11.5	7.5
		**0.002**	SIM	11.5	9.7
**Shh-SBE6**	**4**	**0.011**	SIM	26.3	18.1
**Shh-SBE6**	**5**	**<0.001**	SIM	26.3	13.5
**Shh-SBE6**	**6**	**<0.001**	SIM	26.3	11.0
**Shh-SBE6**	**7**	**0.033**	SIM		
		**0.020**	SIM		
		**0.003**	SIM		
		**0.002**	SIM		
		**<0.001**	SIM	26.3	19,2
		**<0.001**	SIM	11.5	4.3
		**<0.001**	SIM	25.3	9.1
		**<0.001**	SIM	7.8	3.8
		**<0.001**	SIM	15.8	3.9
		**<0.001**	SIM	26	3.6
		**<0.001**	SIM	13.6	4.9
**Shh-SBE4**	**7**	**0.018**	Widefield		
		**0.015**	Widefield		
		**<0.001**	SIM	23.8	6.6
		**<0.001**	SIM	10.6	5.4
**Shh-SBE2/3**	**7**	0.946	SIM		
		0.644	SIM	17.5	18
		0.529	SIM		
		0.493	SIM		
**Shh-ZRS**	**7**	0.057	Widefield		
		0.656	SIM		
		0.594	SIM		
		0.388	SIM		
**Shh-Control**	**7**	0.680	Widefield	Widefield	Widefield
		0.432	SIM		

Table S2. Mann-Whitney U significance tests for Shh-*SBE6 *inter-probe distances between eGFP and TALE-Vp64/TALE-Vp128 transfected cells. Related to Figure 3.

p-values of each biological replicate of the data from Figures 3C and D are shown for the differences between the inter-probe distances from ESCs expressing a control eGFP construct and the data from ESCs expressing TALE-Vp64/Vp128 constructs. Values in bold are significant (p<0.05). Also shown are the % alleles (probe pairs) where probes are considered co-localised (i.e. distances of <200nm).

TALE-Vp64/Vp128 constructs	Shh-SBE6 *p* value	% of probe pair <200nm in eGFP control	% probe pairs <200nm in TALE-fusions
**tShh-Vp128**	0.440	26.6	35.8
	0.073	31.5	8.9
**tSBE6-Vp128**	**0.001**	31.5	**7.5**
**tSBE2-Vp128**	**0.0021**	**22.2**	**1.8**
	**<0.001**	31.5	**0**
**tSBE(6+2)-Vp64**	**0.045**	**8.4**	**5.6**
	**0.013**	**28.3**	**9.6**
	**<0.001**	**32.9**	**3.6**
	**<0.001**	**25.7**	**2.9**
	**<0.001**	**29.5**	**3.5**
	**<0.001**	**31.1**	**18.4**
	**<0.001**	**25.7**	**2.9**
	**<0.001**	**30.8**	**4.1**
**tSBE(6+2)-Δ**	0.228	28.3	28.3

Table S3, Mann-Whitney U significance tests for Shh-*SBE6 *inter-probe distances across the Shh regulatory domain after expression of TALE-LDB1. Related to Figure 3.

Table showing the *p*-values of each biological replicate for differences between the inter-probe distances between the data from ESCs expressing a control eGFP construct and the data from ESCs expressing TALE-LDB1 constructs. Data are from Figures 3H and I. Values in bold are significant (p<0.05). Also shown are the % of alleles (probe pairs) where probes are considered as co-localised (i.e. distances of <200nm) for data from ESCs and cells expressing the various TALE-LBD1 fusions.

Probe pairs	TALE-LDB1 constructs	*p* value	% of probe pair <200nm in ESCs	% of probe pair <200nm in TALE-LBD1
**Shh-SBE6**	**tShh-LDB1 + tSBE6-LDB1**	**0.022**	36.3	54.4
	**tShh-LDB1 + tSBE2-LDB1**	0.457	17.7	21.2
		0.167	22.6	17.2
**Shh-SBE2/3**	**tShh-LDB1 + tSBE2-LDB1**	**0.017**	22.1	34.8
		**<0.001**	18.6	45.5
		**<0.001**	10.5	35.1
**SBE6-SBE2/3**	**tShh-LDB1+tSBE2-LDB1**	0.895	29.1	19.4
		0.198	28.8	29.5
	**tSBE6-LDB1+tSBE2-LDB1**	**0.018**	23.7	48.8

Table S4, Mann-Whitney U significance tests for Shh-SBE6 inter-probe distances after expression of TALE-VWA or SIX3. Related to Figure 4.

p-values of each biological replicate of the data from Figure 4 are shown for the differences between the inter-probe distances from ESCs expressing a control eGFP construct and those from ESCs expressing TALE-VWA or Six3 constructs. Values in bold are significant ($p<0.05$). Also shown are the % alleles (probe pairs) where probes are considered co-localised (i.e. distances of <200nm).

**TALE-constructs**	**Shh-SBE6 *p* value**	**% probe pairs <200nm in eGFP control**	**% probe pairs <200nm in TALE-fusions**
tShh-VWA	0.359	17.4	20.4
tSBE(6+2)-VWA	**0.04**<br>**<0.001**	**17.4**<br>**18.6**	**4.3**<br>**2.6**
tShh-SIX3	0.594	22.6	29.6
tSBE2-SIX3	**0.012**<br>**<0.001**<br>**<0.001**	**22.6**<br>**20.6**<br>**33.3**	**21.4**<br>**8.3**<br>**9.1**

Table S5, Mann-Whitney U significance tests for Shh-SBE6 inter-probe distances after expression of TALEs-that target NE. Related to Figure 5.

p-values of each biological replicate are shown for the differences between the Shh-SBE6 inter-probe distances from ESCs expressing a control eGFP construct and from ESCs expressing various TALE-constructs. Data from Figure 5.

**TALE-constructs**	**Shh-SBE6 *p* value**
tSBE(6+2)-Vp64 + tNE-CTCF	0.626<br>0.602
tSBE(6+2)-Vp64 + tNE-Δ	0.596<br>0.168
tSBE(6+2)-VWA + tNE-Δ	0.627

Table S6. Mann-Whitney U significance tests for Shh-SBE6 inter-probe distances after TALE-fusion expression and after treatment with olaparib. Related to Figure 6.

p-values of each biological replicate are shown for differences between the inter-probe distances from ESCs expressing a control eGFP construct against the data from ESCs expressing TALE constructs, or from NPCs treated with olaparib. Data relate to Figure 6C-F. Values in bold are significant (p<0.05).

**TALE-fusion constructs**	**Shh-SBE6 *p* value**
tSBE6-PARP1	**0.001**
tSBE6-PARP1 + tNE-CTCF	0.106
tSBE2-PARP1	**<0.001**<br>**<0.001**
tSBE2-PARP1 + tNE-CTCF+	0.883
tSBE2-SIX3	**0.012**<br>**<0.001**<br>**<0.001**
tSBE2-SIX3 + Olaparib	0.997<br>0.629<br>0.204
tSBE2-PARP1+Olaparib	0.816<br>0.680<br>0.417
NPC (d7) + Olaparib	0.799<br>0.546
tShh-DEL	**0.003**
tSBE2-DEL	**<0.001**<br>**<0.001**
tSBE2-DEL + Olaparib	**<0.001**<br>**<0.001**

Table S7. Mann-Whitney U significance tests for Shh-SBE6 inter-probe FISH distances across the Shh regulatory domain after recruitment of wild-type and mutant PARP1. Related to Figure 6.

p-values for the differences between the Shh-SBE6 inter-probe distances from ESCs expressing a control eGFP construct and from ESCs expressing various TALE-PARP1 fusion constructs, either wild-type PARP1, or catalytically inactive mutant PARP1s (E988K or D890K+M899N). Data relate to Figure 6H. Values in bold are significant (p<0.05).

**Probe pairs**	**TALE-fusion constructs**	***p* value**
**Shh-SBE6**	tSBE6-PARP1	**<0.001**
	tSBE2-PARP1	**<0.001**
	tSBE6-E988K	0.168<br>0.337
	tSBE6-D890V+M899N	0.794<br>0.226
	tSBE2-E988K	0.756<br>0.447
	tSBE2-D890V+M899N	0.984<br>0.7414

Table S8: Enhancer and FISH probe co-ordinates. Related to STAR Methods section 3D-FISH

The table lists the genome co-ordinates (bp) on chromosome 5 (mm9 assembly of the mouse genome) of *Shh* and its enhancers, and the fosmid probes used for FISH.

Locus	Gene/ enhancer	Fosmid probes			
		start (bp)	end (bp)	fosmid ID	Clone name
CTRL		28411516	28449628	G135P60453C9	WI1-2806K8
Shh	28783380-28793641	28754458	28795879	G135P64333A4	WI1-574O18
SBE6	28889688-28890461	28887686	28924744	G135P67311F4	WI1-442E17
SBE4	29111752-29112865	29107140	29147593	G135P600205H10	WI1-2751A06
SBE2/3	29202018-29219606	29195832	29239355	G135P603171G8	WI1-1275C09
ZRS	29638200-29643599	29611727	29653695	G135P600929F6	WI1-1047E14

Table S9: Table of PCR primers for qRT-PCR and Chip qPCR. Related to STAR Methods section

**qRT-PCR primers**	
Gapdh Fw	ATCACCATCTTCCAGGAGCGAG
Gapdh Rv	GACCCTTTTGGCTCCACCCTTC
Oct4 (Pou5f1) Fw	CGAGAACAATGAGAACCTTC
Oct4 (Pou5f1) Rv	CCTTCTCTAGCCCAAGCTGAT
Nestin Fw	GATCGCTCAGATCCTGGAAG
Nestin Rv	AGGTGTCTGCAAGCGAGAGT
Shh Fw	ACGATTTAAGGAACTCACCC
Shh Rv	TTGTCTTTGCACCTCTGAG
9530036O11Rik Fw	GCCTGAAACACACAGAATGC
9530036O11Rik RV	GATGGGAGAACTCAGCCAAG
Parg Fw	GAAGAAGTGGCTGGGAACTCCT
Parg Rv	GTTTCGGAACCTCTCCTGCTCT
**ChIP qPCR primers**	
tNE Fw	CAAGAAAACATGGGTCTAGGG
tNE Rv	TGAATTGGAACATTTCTTCAGG
tShh Fw	AAGGCTGGAGAGCTTGTGAG
tShh Rv	TAAAGCCACAGCAGCCAGAG
Actin Fw	CCTCGATGCTGACCCTCATCC
Actin Rv	GACACTGCCCCATTCAATGTCTC

Table S10: 5C sequencing technical and biological replicates reads. Related to STAR Methods section

Experiment	No. of reads	No. of used reads	No. of used reads *Shh* reg. region
ESC biological replicate 1 (tSBE-Vp64 experiments)	45,024,284	23,168,046	13,752,662
ESC biological replicate 1	1,745,058	1,368,704	844,392
ESC biological replicate 2	3,606,454	2,830,120	1,751,368
NPC biological replicate 1-technical replicate 1	3,984,330	1,627,144	795,658
NPC biological replicate 1-technical replicate 2	9,814,008	4,168,546	2,267,024
NPC biological replicate 2-technical replicate 1	2,079,276	894,214	411,396
NPC biological replicate 2-technical replicate 2	92,081,330	33,796,970	15,038,310
NPC biological replicate 2-technical replicate 3	16,481,938	5,648,428	2,705,028
tSBE6-Vp64 biological replicate 1	90,452,930	50,035,502	30,790,810
tSBE2-Vp64 biological replicate 1	1.01E+08	44,074,854	21,016,670
tSBE(6+2)-Vp64 biological replicate 1-technical replicate 2	79,792,180	32,329,736	16,415,488
tSBE6-PARP1 biological replicate 1	2,066,022	1,002,156	562,782
tSBE6-PARP1 biological replicate 2	1,664,928	1,255,928	728,276
tSBE6-PARP1 + tNE-CTCF biological replicate 1	2,478,062	1,873,946	1,173,160
tSBE6-PARP1 + tNE-CTCF biological replicate 2	1,604,560	1,231,786	706,104
