# Supplementary material for: Decreased Enhancer-Promoter Proximity Accompanying Enhancer Activation
Source: Mol Cell. 2019 Nov 7;76(3):473–484.e7. doi: 10.1016/j.molcel.2019.07.038 (PMC6838673; doi:10.1016/j.molcel.2019.07.038)
Supplement: Document S2. Article plus Supplemental Information [file mmc2.pdf]

Molecular Cell

Article

Decreased Enhancer-Promoter Proximity Accompanying Enhancer Activation

Graphical Abstract

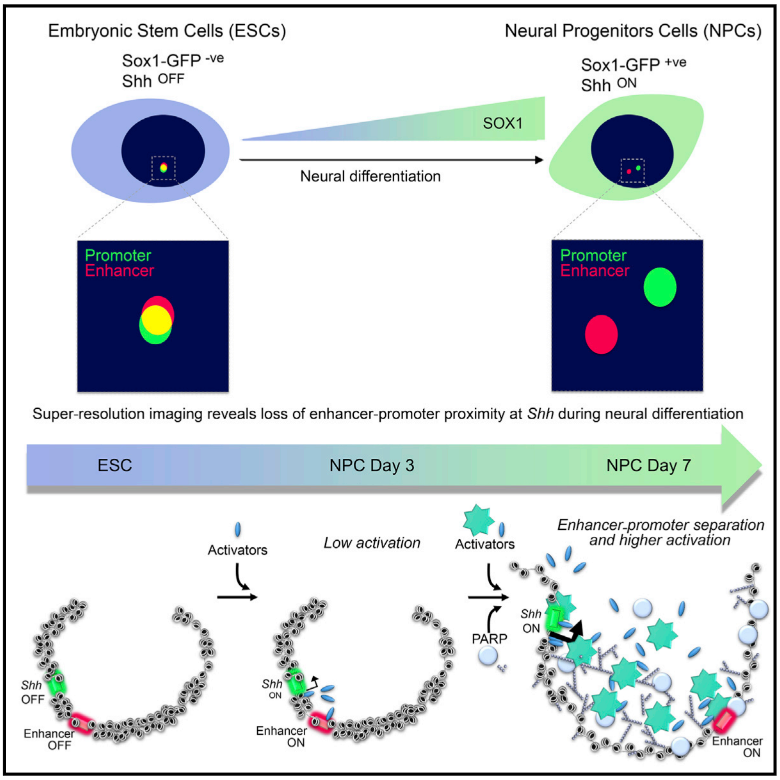

Highlights

- Super-resolution microscopy reveals increased enhancer-promoter separation upon activation
- Synthetic enhancer activation supports decreased enhancer-promoter proximity
- Enhancer-promoter separation can be driven by poly(ADP-ribose) polymerase 1

Authors

Nezha S. Benabdallah, Iain Williamson, Robert S. Illingworth, ..., Graeme R. Grimes, Pierre Therizols, Wendy A. Bickmore

Correspondence

wendy.bickmore@igmm.ed.ac.uk

In Brief

Combining 3D-FISH and chromosome conformation capture, this study monitors long-range chromatin reorganization upon enhancer-driven activation of the Sonic hedgehog gene (*Shh*) during neural differentiation. The increased separation between *Shh* and Shh brain enhancers, which is recapitulated in embryonic stem cells using synthetic activators, seems incompatible with looping models of enhancer function and suggests that other models for enhancer action at a distance need to be considered.

Benabdallah et al., 2019, Molecular Cell *76*, 473–484
November 7, 2019 © 2019 The Author(s). Published by Elsevier Inc.
https://doi.org/10.1016/j.molcel.2019.07.038

CellPress

CellPress

Decreased Enhancer-Promoter Proximity Accompanying Enhancer Activation

Nezha S. Benabdallah,[1,2,4] Iain Williamson,[1] Robert S. Illingworth,[1] Lauren Kane,[1] Shelagh Boyle,[1] Dipta Sengupta,[1] Graeme R. Grimes,[1] Pierre Therizols,[1,3] and Wendy A. Bickmore[1,5,*]
[1]MRC Human Genetics Unit, Institute of Genetics and Molecular Medicine, University of Edinburgh, Crewe Road, Edinburgh EH4 2XU, UK
[2]Edinburgh Super Resolution Imaging Consortium (ESRIC), Institute of Genetics and Molecular Medicine, University of Edinburgh, Crewe Road, Edinburgh EH4 2XU, UK
[3]UMR INSERM 944, CNRS 7212, Bâtiment Jean Bernard, Hôpital Saint Louis, Paris, France
[4]Present address: Pediatric Soft Tissue Sarcoma Research Group, Hopp Children's Cancer Center Heidelberg (KiTZ), German Cancer Research Center (DKFZ), Heidelberg, Germany
[5]Lead Contact
*Correspondence: wendy.bickmore@igmm.ed.ac.uk
https://doi.org/10.1016/j.molcel.2019.07.038

SUMMARY

Enhancers can regulate the promoters of their target genes over very large genomic distances. It is widely assumed that mechanisms of enhancer action involve the reorganization of three-dimensional chromatin architecture, but this is poorly understood. The predominant model involves physical enhancer-promoter interaction by looping out the intervening chromatin. However, studying the enhancer-driven activation of the Sonic hedgehog gene (*Shh*), we have identified a change in chromosome conformation that is incompatible with this simple looping model. Using super-resolution 3D-FISH and chromosome conformation capture, we observe a decreased spatial proximity between *Shh* and its enhancers during the differentiation of embryonic stem cells to neural progenitors. We show that this can be recapitulated by synthetic enhancer activation, is impeded by chromatin-bound proteins located between the enhancer and the promoter, and appears to involve the catalytic activity of poly (ADP-ribose) polymerase. Our data suggest that models of enhancer-promoter communication need to encompass chromatin conformations other than looping.

INTRODUCTION

Enhancers are *cis*-regulatory sequences, often located within the non-coding portion of the genome, which regulate spatial and temporal gene expression in development and physiology. Enhancers can operate when located proximal to or very distant (100s–1000s of kb) from their target gene (Vernimmen and Bickmore, 2015). Well-established molecular signatures of active enhancers include clustered sequence-specific transcription factor (TF) binding sites and DNase I hypersensitive (DHS) sites, specific histone modifications (e.g., H3K4me1, acetylation of specific lysine residues on histone H3 [H3K27ac, H3K64ac, H3K122ac] and H4 [H4K16ac]), and, in some cases, enhancer RNA (eRNA) transcription (Kim et al., 2010; Pradeepa et al., 2016; Shlyueva et al., 2014; Taylor et al., 2013). Less is known about the mechanisms by which enhancers communicate with and control the expression of their target gene promoter(s).

For proximal enhancers, it has been proposed that activation signals nucleated by bound TFs can move toward the target gene by facilitated diffusion or by tracking along the intervening chromatin, even modifying the intervening chromatin along the way (Bulger and Groudine, 2011; Benabdallah and Bickmore, 2015). These models have been considered unlikely as mechanisms for more distal enhancers.

For very-long-range regulation, communication between the enhancer and the promoter is generally thought to occur through the interaction of protein complexes bound at both sites, with looping out of the intervening chromatin. Chromatin "looping" has been best illustrated for interactions between the β-globin gene and its locus control region (LCR); enhancer-promoter interactions have been detected by chromosome conformation capture (3C) methods (Carter et al., 2002; Tolhuis et al., 2002), and experimentally forced enhancer-promoter chromatin looping can contribute to transcriptional activation (Bartman et al., 2016; Deng et al., 2012, 2014; Morgan et al., 2017). Using fluorescence *in situ* hybridization (FISH), we have visualized the spatial juxtaposition of a target gene (*Shh*) with its distant (1 Mb) limb enhancer (ZRS), with a looping out of the intervening chromatin, specifically in *Shh*-expressing tissue of the developing limb bud (Williamson et al., 2016). However, the generality of enhancer-promoter looping remains unclear, and other chromatin conformations may contribute to long-range gene regulation from enhancers (Benabdallah and Bickmore, 2015; Brown et al., 2018). Live cell imaging also fails to provide evidence for enhancer-promoter spatial proximity driving transcription in mouse embryonic stem cells (mESCs) (Alexander et al., 2019).

The Sonic hedgehog morphogen (Shh) governs the growth and patterning of many tissues during development. Precise spatial and temporal control of *Shh* expression is regulated by tissue-specific enhancers located within the introns of the gene, upstream of the *Shh* transcription start site (TSS) in a large (750 kb) gene desert

This is an open access article under the CC BY license (http://creativecommons.org/licenses/by/4.0/).

and within genes at the far end of the gene desert (Anderson and Hill, 2014). *Shh* expression is important for several aspects of brain development. Shh-Brain-Enhancers-6 (SBE6), SBE2/3, SBE4, and SBE5, located $\geq$100 kb upstream of the *Shh* TSS, drive expression in the midbrain and anterior domains of the developing brain (Benabdallah et al., 2016; Jeong et al., 2006; Yao et al., 2016). Here, we observe that during the induction of *Shh* expression in neural progenitor cells (NPCs) or *in vivo* in the *Shh*-expressing cells of the embryonic neural tube, there is a decrease in enhancer-promoter proximity that is not compatible with an enhancer-promoter looping model. We show that synthetic enhancer activation in mESCs also leads to enhanced separation between *Shh* and SBE6, SBE4, or SBE2/3 enhancers. Our data suggest a role for polyADP-ribosylation in this increased spatial separation of enhancers and promoters, and we discuss these findings in the context of new biophysical models of enhancer function.

RESULTS

Decreased Shh and Shh-Brain-Enhancers Co-localization upon Neural Differentiation

As the known *Shh*-Brain-Enhancers SBE5, SBE2/3, SBE4, and SBE6 are located 780, 450, 350, and 100 kb upstream of *Shh,* respectively (Figure 1A), the present models assume that these enhancers would physically loop to the *Shh* promoter in neural cells and tissues. We have previously used super-resolution microscopy, in conjunction with three-dimensional (3D)-FISH, to demonstrate the spatial juxtaposition of *Shh* and its limb enhancer (ZRS), with displacement of an intervening genomic region, restricted to the time and place of *Shh* expression in the developing limb (Williamson et al., 2016).

To analyze the spatial relation of *Shh* to its known brain enhancers, we differentiated 46c mESCs (Ying et al., 2003) into NPCs, monitoring differentiation by *Sox1*-GFP fluorescence (Benabdallah et al., 2016). We performed 3D-FISH on mESCs that do not express *Shh* and on NPCs obtained after 7 days of differentiation, when *Shh* is expressed (Benabdallah et al., 2016), and we imaged the slides by 3D-structured illumination microscopy (3D-SIM). Within the *Shh* domain, the most prominent peaks of H3K4me1 and H3K27ac gained during differentiation occur at SBE6, an enhancer required for the full induction of *Shh* during this NPC differentiation program (Benabdallah et al., 2016). Upon *Shh* activation, there was a significant increase in inter-probe distances between *Shh* and both SBE6 and SBE4, located 100 and 350 kb 5′ of *Shh,* respectively, and a decrease in the proportion of co-localized alleles (enhancer-promoter inter-probe distances $\leq$0.2 μm) (Figures 1B and 1C; Table S1). For example, for SBE6-Shh, the proportion of alleles with inter-probe distances $\leq$0.2 μm ranged between 7.8% and 26.3% in mESCs between replicate experiments and fell to 3.6%–19.2% in NPCs (Table S1). For SBE4-Shh, the ranges were 10.6%–23.8% in ESCs and 5.4%–6.6% in NPCs. Distances between *Shh* and the more distant ZRS or a control probe (CTRL) located outside the *Shh* regulatory domain were not significantly changed (Figures 1A and 1B; Table S1).

The decreased proportion of alleles with enhancer-promoter juxtaposition upon *Shh* induction does not seem compatible with the formation of a chromatin loop between *Shh* and the SBE6/SBE4/SBE2 neural enhancers. To assess whether looping occurs at an earlier time point, we analyzed *Shh* expression during the NPC differentiation time course (days 3–7; D3–D7). Some *Shh* expression initiated on D3, increasing steadily until D6 or D7 (Figure 1D). In all of the replicate experiments, Shh-SBE6 separation increased significantly from D4 onward. Shh-SBE6 distances were somewhat increased at D3, but only reached statistical significance for two of the three biological replicates (Figures 1E and S1A). These data support the notion that no stable chromatin loops are formed between SBE6 and Shh at an earlier time point during this neural differentiation program (Table S1).

Single-cell qRT-PCR showed that there is increased *Shh* expression in most cells of the population at D3 and D4 of NPC differentiation (Figures 1F and S1B), and even higher levels by D7, consistent with the cell population averaged expression data (Figure 1D). This excludes that there is a small subpopulation of NPCs that express *Shh* at high levels, possibly with a looped chromatin conformation. For the same cell populations, Shh-SBE6 inter-probe distances start to increase at D3–D4 and shift homogenously toward greater distances at D5–D7 (Figures 1E and S1A). There is no statistical evidence for bimodality in the data distribution.

No Increased Enhancer-Promoter Co-localization *In Vivo*

Shh is expressed in ventral regions of the neural tube—in the floor plate and notochord (Jeong et al., 2006) (Figure 2A)—and transgene assays indicate that SBE6 is also active in the floor plate (Benabdallah et al., 2016). To assess SBE6-*Shh* proximity *in vivo*, we used FISH to examine *Shh*-SBE6 inter-probe distances in sections through the neural tube of an E10.5 embryo. RNA FISH indicated that 49% of *Shh* alleles are expressed in this region of the neural tube (Figure 2A). DNA FISH showed that *Shh*-SBE6 inter-probe distances were greater, and the proportion of alleles with enhancer-promoter co-localization lower (9.9%), in nuclei from the floor plate region (9.9%) compared to dorsal neural tube cells (21.9%) (Figures 2B and 2C). This suggests that there is no elevated enhancer-promoter proximity in *Shh*-expressing cells *in vivo* during neurogenesis.

To determine whether other as yet unidentified *cis*-regulatory elements gain interactions with the *Shh* promoter during the differentiation of ESCs to NPCs, we used chromosome conformation capture carbon copy (5C) to assay cross-linked ligation frequencies across the entire *Shh* regulatory domain. Consistent with Hi-C data from ESCs (Smallwood and Ren, 2013) and 5C data from E11.5 embryos (Williamson et al., 2016), in both ESCs and NPCs, all of the SBEs are contained in a topologically associated domain (TAD) that extends from downstream of *Shh* (before *Rbm33*) to just beyond *Lmbr1*, the gene where the ZRS is located. Comparison of 5C data from ESCs and D7 NPCs revealed no evidence for a gain of specific interactions in NPCs that may indicate the formation of a loop between *Shh* and its neural enhancers (Figures 2D and S2A).

SBE Activation Increases Enhancer-Promoter Separation

Supercoiling associated with transcription decondenses large chromatin domains (Naughton et al., 2013); therefore, the altered

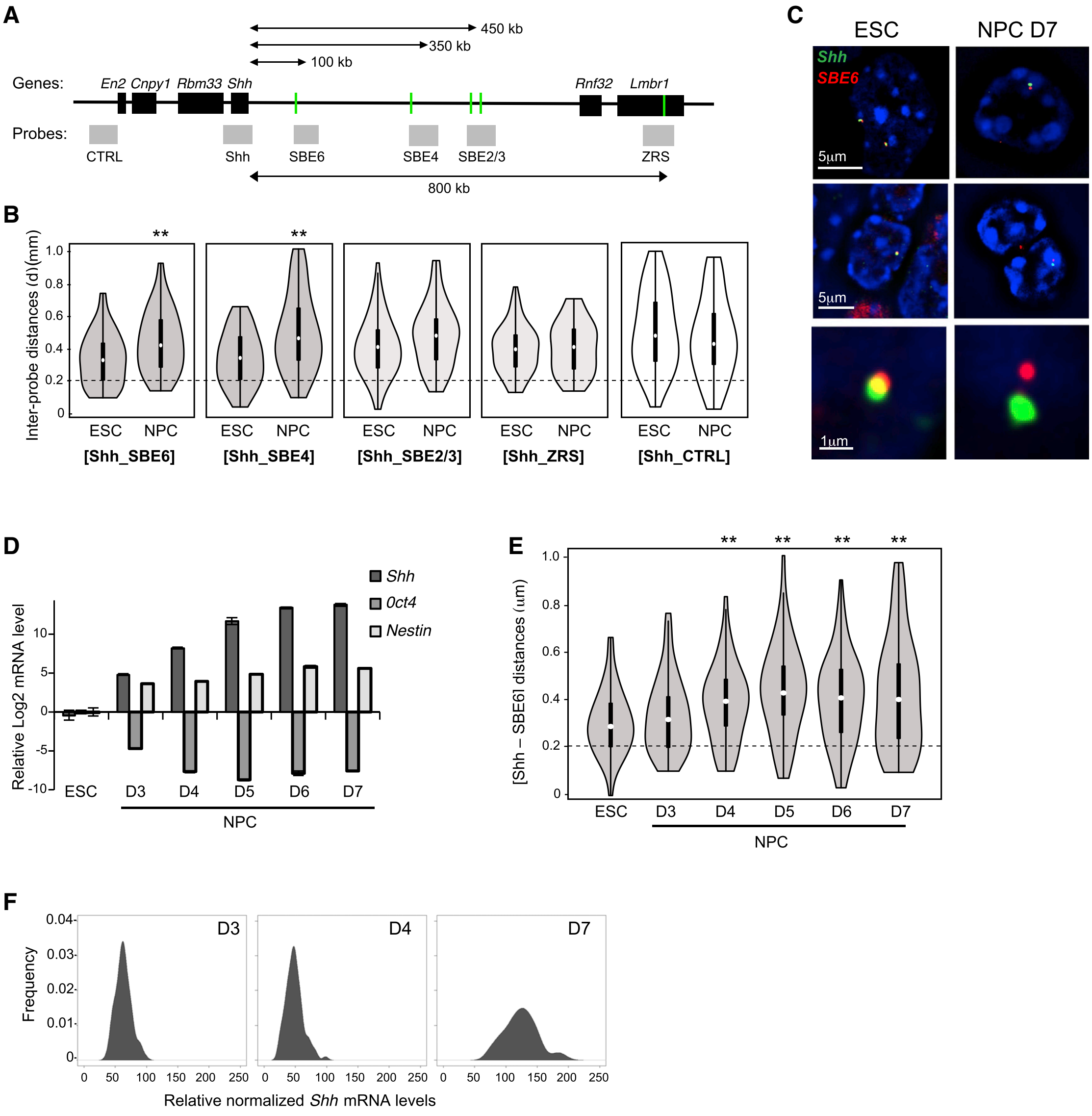

Figure 1. Loss of Shh-Brain-Enhancer Proximity during Neuronal Differentiation

(A) Map of the *Shh* regulatory domain showing the genes (black boxes), enhancers (green bars), and fosmid FISH probes (gray boxes). Probe and enhancer coordinates are listed in Table S8.

(B) Violin plots showing the distribution of inter-probe distances (μm) between *Shh* and SBE6, SBE4, SBE2/3, ZRS, and CTRL probes in the nuclei of ESCs and D7 NPCs. Distances below the dotted horizontal line at 0.2 μm are considered co-localized. The asterisks on the FISH data represent Mann-Whitney *U* test significance between ESC and NPC populations. **$p < 0.01$. Each violin plot represents one biological replicate; for other replicates and statistics, see Figure S1 and Table S1.

(C) 3D-SIM images illustrating *Shh*-SBE6 separation in ESCs or in D7 NPCs. Scales bars are 5 μm (top two rows) and 1 μm (bottom, inset from center row).

(D) *Shh*, *Oct4*, and *Nestin* expression assayed by qRT-PCR during a time course of NPC differentiation. The graph shows mean (±SEM) log2 mRNA levels relative to *Gapdh* and normalized to the level in ESC (three technical replicates).

(E) Violin plots showing *Shh*-SBE6 inter-probe distances in cell populations corresponding to the expression data in (D).

(F) Kernel density plots showing *Shh* mRNA expression in single NPCs relative to *Gapdh* and normalized to the expression in ESC. Density is an arbitrary unit based on the frequency of the occurrence and the total counts and the size of the population (i.e., the binning of the data).

Data from a biological replicate are shown in Figure S1B.

chromatin conformations we observe during NPC differentiation could occur as a passive consequence of *Shh* transcription. To test this, we bypassed the need for Shh enhancers, fusing a transcription activator-like effector (TALE) targeted to the *Shh* promoter (tShh) to repeats of the small viral acidic protein VP16 (VP64/VP128) that can strongly activate gene expression (Zhang et al., 2011) (Figure 3A), including in ESCs (Therizols et al., 2014). The expression of tShh-VP64/128 in mESCs led to the activation of *Shh* expression to levels similar to those seen in NPCs (Figure 3B), but without perturbing markers of pluripotency or

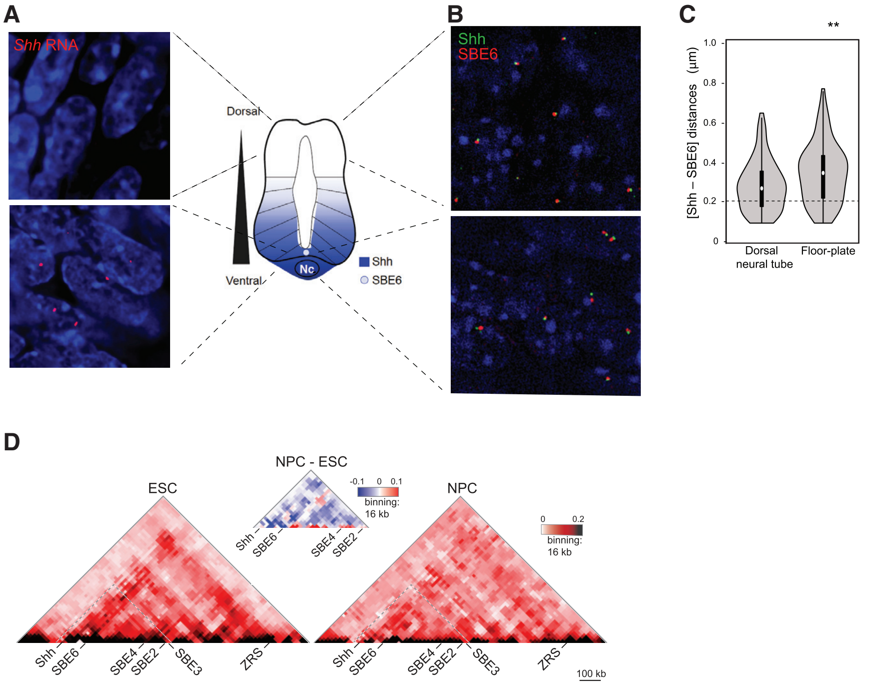

Figure 2. Enhancer-Promoter Separation Occurs *In Vivo*

(A) Schematic of a transverse section through the neural tube with a gradient of Shh emanating from ventral *Shh*-expressing cells in the notochord (Nc) and the floor plate, where SBE6 activity is also detected (Benabdallah et al., 2016). Panels at left show RNA FISH signal (red) for *Shh* in sections from the dorsal neural tube (top panel) or the floor plate (bottom panel) of E10.5 mouse embryos.

(B) 3D-FISH SIM images illustrating *Shh*-SBE6 separation in nuclei from the dorsal neural tube (top) or ventral floor plate (bottom) of E10.5 mouse embryos.

(C) Violin plots showing Shh-SBE6 inter-probe distances for FISH data from E10.5 dorsal neural tube and ventral floor plate cells. **$p < 0.01$ for this biological replicate. For two other biological replicates, $p = 0.002$ and $p < 0.001$.

(D) 5C heatmaps of the *Shh* regulatory region (mm9, chr5:28604000-29780000) from ESCs and D7 NPCs with 16 kb binning and smoothing. A difference plot for the 5C heatmaps between NPCs and ESCs is shown above.

Data from a biological replicate are shown in Figure S2C.

neuronal differentiation (Figure S3A). Synthetic activation was restricted to *Shh* and the long non-coding RNA *9530036O11Rik* that is transcribed in the opposite direction from the *Shh* promoter (Figures S3B and S4C). Like *Shh*, *9530036O11Rik* expression is also induced during NPC differentiation. The two other genes in the Shh TAD (*Rnf32* and *Lmbr1*) are not activated during NPC differentiation or by TALE-VP16 targeting to the *Shh* promoter or SBE6. The same is true of two genes flanking the *Shh* TAD (*Rbm33* and *Nom1*) (Figure S3B).

Synthetic activation of *Shh* using promoter-targeted VP16 did not alter *Shh*-SBE6 inter-probe distances (Figure 3C; Table S2). Therefore, the increased enhancer-*Shh* promoter separation observed during NPC differentiation is not simply a consequence of activating *Shh* or *9530036O11Rik* expression.

Recruiting VP64/128 to SBE2 or SBE6 also induced *Shh* expression, albeit less markedly compared to direct recruitment to the *Shh* promoter (Figure 3B), and without detectable effects on the expression of the pluripotency or neuronal markers tested (Figure S3A). However, VP128 recruitment to either enhancer resulted in an increase in enhancer-promoter separation (Figure 3C) and decreased enhancer-promoter proximity—the percentage of alleles with inter-probe distances <200 nm decreasing from 22%–32% in eGFP controls to <7.5% in SBE-VP128-expressing transfectants (Table S2). A similar result was achieved by recruiting VP64 to SBE6 and SBE2 simultaneously: tSBE(6+2)-VP64 (Figures 3B and 3D; Table S2). Decreased enhancer-promoter proximity was specific to VP64 activity as recruiting TALEs without a fusion domain (tSBE(6+2)-Δ) had no effect (Figure 3D; Table S2). 5C analysis (Figure 3E) and virtual 4C analysis of that data (Figure S3C) also revealed a loss of interactions between the activated enhancers and the *Shh* promoter and with other sequences in the *Shh*-SBE2 interval.

To show that FISH is capable of detecting enhancer-promoter proximity as a result of chromosome looping, we created artificial Shh-SBE interactions. Targeted tethering (using zinc fingers) of the self-association (SA) domain of LIM domain-binding protein 1 (LDB1) has been used previously to force a chromatin loop at the β-globin locus (Bartman et al., 2016; Deng et al., 2012, 2014). Using a similar approach, but with TALE proteins, we tethered the LBD1 SA to the *Shh* promoter (tShh-LDB1) and to either SBE6 or SBE2 (tSBE6-LDB1 and tSBE2-LDB1) in mESCs (Figure 3F). The configurations of FISH probe signals were consistent with the predicted loop (Figure 3G). Quantification revealed dramatically increased co-localization (35%–50% of alleles at ≤200 nm) between the tethering sites upon tShh-LDB1 and tSBE6/tSBE2-LDB1 co-transfection (Figures 3H and 3I; Table S3). In cells transfected with tShh-LDB1 and tSBE2-LDB1, SBE6 was further from *Shh* than was the genomically more distant SBE2/3 (Figure 3I), which is consistent with a chromatin loop anchored by LDB1 interactions. We conclude that 3D FISH is able to detect a chromatin loop in ESCs, albeit an artificially constructed one.

Endogenous Activators and Co-activators Also Reduce Enhancer-Promoter Proximity

VP16 is a very effective transcriptional activator, but of viral origin. We therefore wished to analyze whether mammalian endogenous activators and co-activators could induce similar alterations in enhancer-promoter proximity. The Mediator complex is recruited to active enhancers and promoters (Soutourina, 2018) and can work alongside cohesin to alter 3D chromosome conformation upon enhancer-driven gene activation (Kagey et al., 2010; Phillips-Cremins et al., 2013). Chromatin immunoprecipitation (ChIP) showed that Mediator is recruited to the

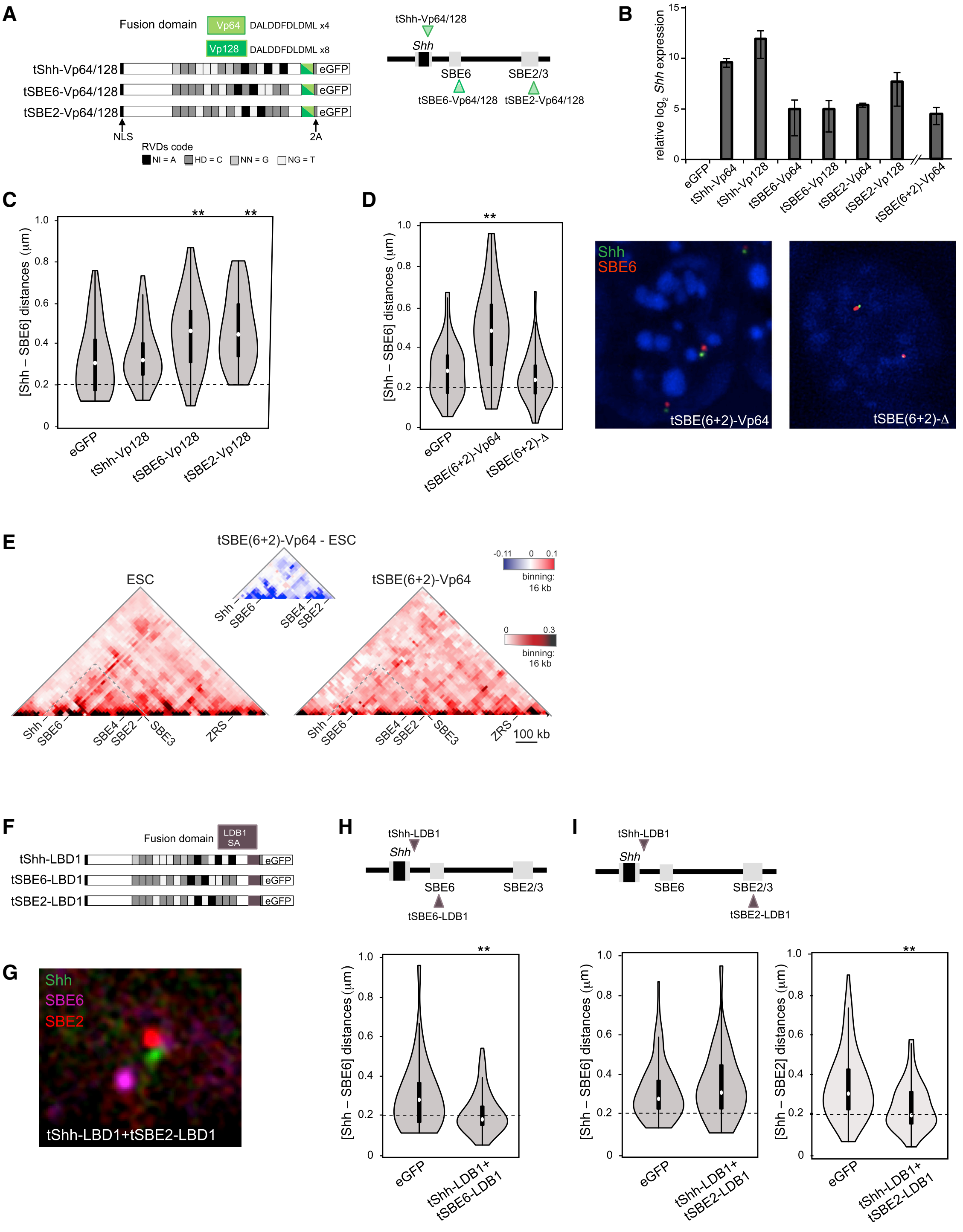

Figure 3. Synthetic Activation of Shh and Increased Enhancer-Promoter Separation Using TALE-VP16

(A) Schematic of TALE-VP64 and TALE-VP128 constructs targeting the *Shh* promoter (tShh), SBE6, or SBE2. Repeat variable diresidue (RVD) code is displayed with one-letter abbreviations for amino acids. Self-cleaving (2A) peptide allows the expression of eGFP and cell isolation by fluorescence-activated cell sorting (FACS). A map of the targeting sites is shown at right.

(B) Log2 mRNA levels of *Shh,* relative to *Gapdh,* assayed by qRT-PCR after TALE-VP64/128 expression in ESCs. Data show means (±SEMs) of three biological replicates normalized to ESCs expressing a control eGFP.

(legend continued on next page)

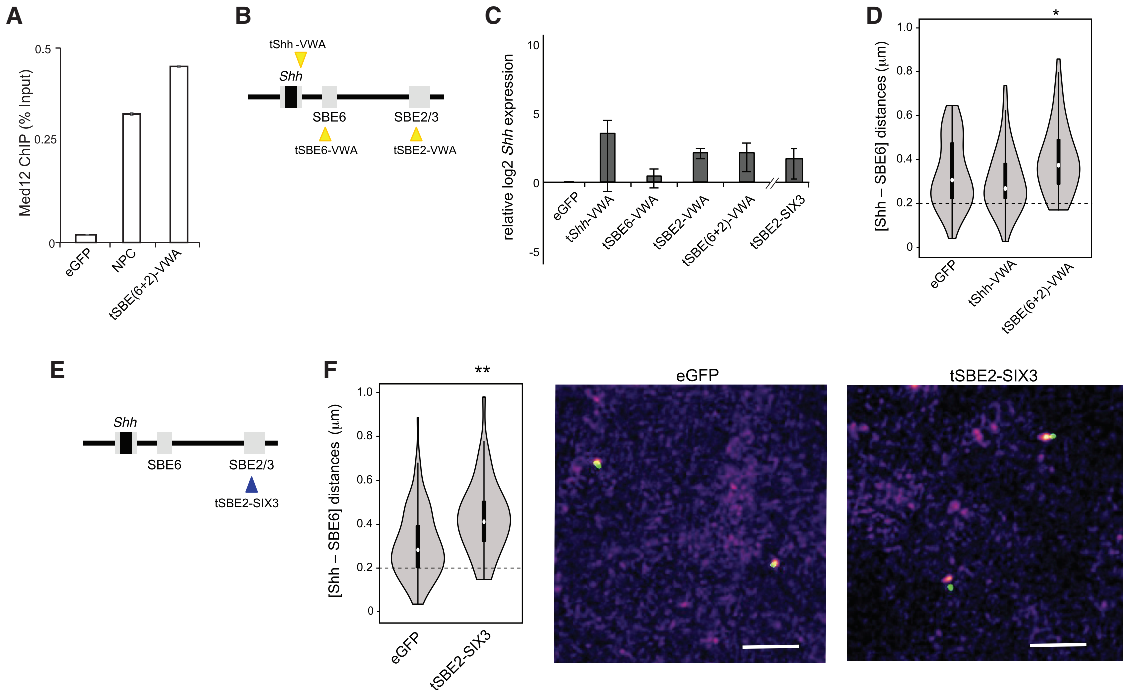

Figure 4. Enhancer-Promoter Separation Induced by Endogenous Activators and Co-activators

(A) Med12 ChIP (percentage of input, normalized to β-actin promoter) at *Shh* promoter measured by qPCR in NPC or in ESCs expressing eGFP or tSBE(6+2)-vWA.

(B) Schematic showing the targeting of TALE-vWA constructs.

(C) Log2 mRNA levels of *Shh* relative to *Gapdh* in ESCs expressing TALE-vWA constructs targeting the *Shh* promoter (tShh), SBE6, SBE2, or both SBE6 and SBE2. Data for a TALE targeting SIX3 to SBE2 are also shown. Data show means (±SEMs) of three biological replicates normalized to ESCs expressing eGFP.

(D) Violin plots of Shh-SBE6 inter-probe distances (μm) in cells expressing eGFP or TALE-vWA targeting the *Shh* promoter (tShh), or both SBE6 and SBE2. *p = 0.018. Statistical data and replicate experiments are shown in Table S4.

(E) Schematic showing the targeting of TALE-SIX3 construct to SBE2.

(F) As in (D), but for ESCs expressing tSBE2-SIX3. **p < 0.01. Example FISH images are shown at right. Bar, 2 μm.

Shh promoter during the differentiation of 46c ESCs to NPCs (Figure 4A).

Mediator interacts with RNA polymerase II and many transcription factors and is believed to bridge between them. VP16 interacts with the Med25 subunit located in the tail domain of the complex (Milbradt et al., 2011; Vojnic et al., 2011), and Med25 is recruited into the Mediator complex through its N-terminal von Willebrand factor A (vWA) domain (Mittler et al., 2003). We therefore fused TALEs to the Med25 vWA domain (Figure 4B). Compared to TALE-VP16, Med25-vWA recruitment induced only low-level *Shh* expression, even for the *Shh* promoter-targeted TALE (tShh-vWA) (Figure 4C). Recruitment of vWA to the *Shh* promoter did not alter chromatin conformation upstream of *Shh* (Table S4). Targeting to both SBE6 and SBE2 (tSBE(6+2)-vWA) resulted in the recruitment of the Med12 subunit of the Mediator kinase module to the *Shh* promoter, compatible with a long-range effect (Figure 4A); as for VP64 recruitment, it led to increased promoter-enhancer separation, and the frequency of co-localized (≤200 nm) SBE6-Shh alleles decreased from 17.4%–18.6% in eGFP controls to 2.6%–4.3% (Figure 4D; Table S4).

The endogenous TFs that bind and activate SBEs in neural tissues are largely unknown. However, Six homeobox 3 (SIX3) is

(C) Violin plots representing one biological replicate of Shh-SBE6 inter-probe distances (μm) in ESCs expressing control eGFP, TALE-VP128 fusions targeting *Shh* promoter (tShh), SBE6, or SBE2. **p < 0.01. Statistical data and replicate experiments are in Table S2. Distances below the dotted horizontal line at 0.2 μm are considered co-localized.

(D) As in (C), but for VP64 recruitment to both SBE6 and SBE2 simultaneously (tSBE6+2), or a TALE with no fusion protein (tSBE6+2)-Δ. **p < 0.01. Representative FISH images with probes for Shh (green) and SBE6 (red) in mESCs expressing tSBE(6+2)-VP64 and tSBE(6+2)-Δ are shown at right.

(E) 5C heatmaps of the *Shh* regulatory region (chr5:28604000-29780000) with 16 kb binning and smoothing for ESCs and for ESCs expressing TALE-VP64 fusions targeting both SBE6 and SBE2. Difference 5C plots are shown above the main 5C plots.

(F) Schematic representing TALE-LDB1 targeting sequences.

(G) Three-color FISH with probes for Shh (green), SBE6 (magenta), and SBE2 (red) in mESCs expressing tShh-LDB1+tSBE2-LDB1.

(H) Violin plots displaying *Shh* and SBE6 inter-probe distances (μm) in ESCs expressing eGFP or tShh-LDB1+tSBE6-LDB1. **p < 0.01.

(I) As in (H), but in cells expressing tShh-LDB1+tSBE2-LDB1. Shh-SBE6 distances are shown in the left-hand panel, and Shh-SBE2 distances are in the right-hand panel. **p < 0.01.

Statistical data relating to this figure are included in Table S3.

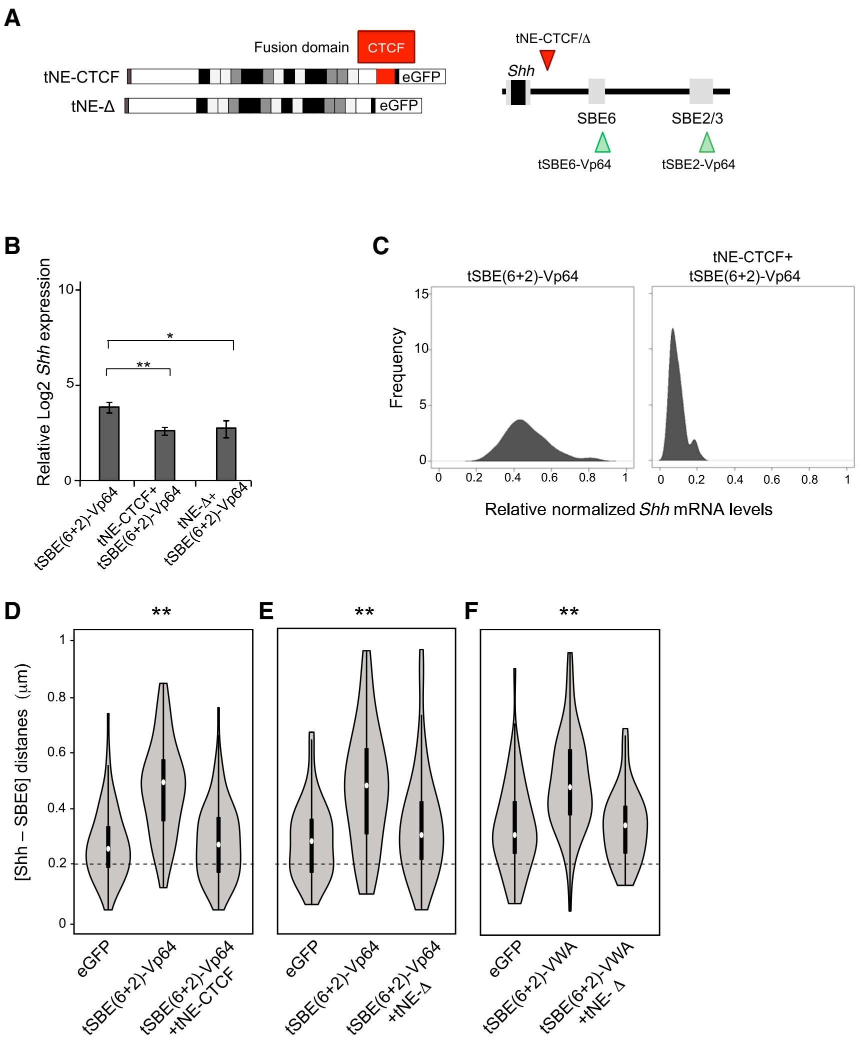

Figure 5. Enhancer-Promoter Spatial Separation Is Blocked by Intervening Chromatin-Bound Proteins

(A) Schematic showing TALEs targeting the NE site with either no fusion protein (tNE-Δ) or fused to CTCF (tNE-CTCF).

(B) Log2 mean (±SEM) *Shh* mRNA levels relative to *Gapdh* in ESCs expressing TALE-VP64 fusions targeting SBE6 and SBE2 and in cells that also express either tNE-CTCF (three biological replicates) or tNE-Δ (four biological replicates). Data are normalized to those from ESCs expressing control eGFP. The asterisks represent p values for a one-tailed Student's t test between conditions. $^*p < 0.05$ and $^{**}p < 0.01$.

(C) Kernel density plots showing *Shh* expression in single ESCs expressing TALE-VP64 fusions targeting SBE6+SBE2 and in these cells when tNE-CTCF is also expressed. Expression is normalized to that in ESCs.

(D and E) Violin plots representing one biological replicate of Shh-SBE6 inter-probe distances (μm) in ESCs expressing eGFP, TALE-VP64 fusions targeting SBE6+SBE2, and these cells when either tNE-CTCF (D) or tNE-Δ (E) is also expressed.

(F) As in (E), but using a TALE-vWA fusion targeting SBE6 and SBE2. $^*p < 0.05$ and $^{**}p < 0.01$.

Statistical data for FISH data from this figure are shown in Table S5.

known to bind to SBE2, and mutation of its binding site, or of SIX3 itself, affects *Shh* expression in the developing brain, leading to holoprosencephaly (HPE) (Geng et al., 2008; Jeong et al., 2008). *Six3* is also upregulated during the *ex vivo* differentiation of ESCs to NPCs (Benabdallah et al., 2016). This prompted us to investigate whether tethering SIX3 was sufficient to recapitulate chromatin conformation changes in the region 5′ of *Shh*. TALE-directed recruitment of SIX3 to SBE2 (Figure 4E) induced only very low-level and variable *Shh* expression (Figure 4C), but nevertheless led to increased inter-probe distances 5′ of *Shh* (Figure 4F; Table S4). Therefore, recruitment of either an endogenous activator (SIX3) or a co-activator (Mediator) to enhancers 5′ of *Shh* leads to increased enhancer-promoter separation.

Intervening Proteins Abrogate the Loss of Enhancer-Promoter Proximity

The absence of detectable enhancer-promoter juxtaposition upon *Shh* activation *in vitro* and *in vivo* and evidence of increased enhancer-promoter separation in these conditions seem incompatible with chromatin-looping mechanisms for enhancer action, but they may be more consistent with spreading and/or linking or tracking-like models (Bulger and Groudine, 2011; Engel et al., 2008; Vernimmen and Bickmore, 2015). To investigate this further, we attempted to insert obstacles between SBE6 and *Shh*. We chose a site 65 kb upstream of the *Shh* TSS (chr5:28859721; mm9) that lacks evidence both of enhancer activity (H3K4me1/H3K27ac marks) during ESC-NPC differentiation (Benabdallah et al., 2016) and evolutionary conservation. We fused a TALE construct specific to this site (NE [Non-Enhancer]) to CTCF (tNE-CTCF) (Figure 5A), as CTCF has been proposed to have general enhancer-blocking functions (Burgess-Beusse et al., 2002) and to block enhancer-promoter tracking (Engel et al., 2008). Transfection of tNE-CTCF, in conjunction with TALE-VP64 co-activation of SBE6 and SBE2, reduced *Shh* activation in the cell population (Figure 5B), and single-cell qRT-PCR confirmed that the majority of the cells transfected with tNE-CTCF+tSBE(6+2)-VP64 had low levels of *Shh* expression (Figure 5C). tNE-CTCF also prevented the increase in enhancer-promoter separation induced by the TALE-VP64 co-activation of SBE6 and SBE2 (Figure 5D; Table S5), which is consistent with the intervening CTCF molecule interrupting a mechanism initiated at SBE6/2. As a control, we also recruited a TALE without any fused protein (tNE-Δ) (Figure 5A). The introduction of tNE-Δ also abrogated *Shh* induction (Figure 5B) and the increased enhancer-promoter separation induced by tSBE(6+2)-VP64 or tSBE(6+2)-vWA (Figures 5E and 5F; Table S5).

H3K27me3, H3K27ac, and Transcription Are Not Mediating Decreased Enhancer-Promoter Proximity at Shh

A class of poised enhancers in mESCs are marked by H3K27me3 (Rada-Iglesias et al., 2011), and our data may therefore result from a loss of long-range interactions between such poised enhancers and *Shh* upon differentiation or synthetic gene activation. However, H3K27me3 ChIP profiling in mESCs (Illingworth et al., 2015) showed that whereas the *Shh* gene itself is a target of polycomb, there are no other blocks of H3K27me3 in the *Shh* regulatory region that could indicate the presence of additional repressors or poised enhancers (Figure S4A).

There are examples in which histone acetylation spreads between an enhancer and a target gene and is blocked by CTCF (Zhao and Dean, 2004). ChIP showed that *Shh* activation using TALE-VP64 constructs targeted to SBE6 or SBE2 induced histone acetylation (H3K27ac), but this was limited precisely to the TALE binding site, with no indication of spreading along the intervening chromatin to *Shh* (Figure S4B). Similarly, RNA polymerase II has been demonstrated to track between some enhancer and promoter regions, synthesizing short poly-adenylated RNAs (Zhu et al., 2007). The expression of the long non-coding RNA *9530036O11Rik* on the opposite strand from *Shh* is also upregulated during neural differentiation (Figures S3B and S4C). However, as *9530036O11Rik* transcription is also induced by targeting VP16 to the *Shh* promoter (Figure S3B), this does not account for the increase in inter-probe separation between *Shh* and SBE6 (Figure 3C).

Assaying nascent transcription in mESCs and during NPC differentiation using 4-thiouridine (4sU) sequencing also provided no evidence for any additional transcription, apart from *Shh* and *9530036O11Rik*, induced across the *Shh* intergenic region on either strand during NPC differentiation (Figure S4C).

Increased Enhancer-Promoter Separation Appears to Involve Poly(ADP-Ribosyl)ation

Because poly(ADP-ribosyl)ation (PARylation) catalyzed by poly(ADP-ribose) polymerases has been linked to large-scale changes in chromatin structure such as decompaction (Huletsky et al., 1989; Poirier et al., 1982), with chromatin remodeling (Singh et al., 2017) and gene activation at ecdysone and heat shock-induced puffs on *Drosophila* polytene chromosomes (Sawatsubashi et al., 2004; Tulin and Spradling, 2003; Tulin et al., 2003), we wanted to determine whether it could be involved in the localized changes in chromosome conformation at Shh. We therefore used TALEs to investigate whether targeting PAR polymerase 1 (PARP1) to *Shh*, SBE6, or SBE2 altered chromatin conformation at the *Shh* region (Figure 6A). PARP1 recruitment had a minimal effect on *Shh* expression (Figure 6B), but it led to an increased *Shh* and SBE6 separation when targeted to SBE6 or SBE2 (Figure 6C; Table S6). This could be blocked by co-transfection with tNE-CTCF (Figure 6C), as was observed with TALE-VP16 constructs (Figure 5D). Similarly, 5C analysis showed a relative loss of interactions in the region 5′ of *Shh* when Parp1 was recruited to SBE6 (Figure 6G), and these interactions were restored when CTCF was tethered to NE between SBE6—the site of PARP1 recruitment—and *Shh* (Figures 6G and S5).

To assess whether the increased enhancer-promoter spatial separation seen by targeted recruitment of other activators such as SIX3 (Figures 4G and 5H) could also involve PARP1 catalytic activity, we used the PARP inhibitor olaparib. Olaparib treatment prevented the *Shh*-SBE6 distance increases mediated by either SIX3 or PARP1 recruitment to SBE2 (Figure 6D) and those seen upon the differentiation of mESCs to NPCs (Figure 6E; Table S6). The effect of olaparib is not a generic effect on chromatin conformation. Visible chromatin decompaction could also be induced by recruiting the small acidic peptide DELQPASIDP (DEL) to SBE2. This peptide decompacts chromatin without leading to gene activation (Carpenter et al., 2005; Therizols et al., 2014). Olaparib had no effect on the increased enhancer-promoter distances induced by DEL recruitment (Figure 6F).

We further tested a requirement for PARP1 catalytic activity using TALE-mediated recruitment of PARP1 E988K and M890V+D899N, which are mutations in the catalytic domain of PARP1 (Rolli et al., 1997). In contrast to wild-type PARP1, recruitment of mutant PARP1 to SBE2 or SBE6 did not alter *Shh*-SBE6 distances (Figure 6H; Table S7). We conclude that most of the changes in chromatin structure that we have detected as a result of recruitment of activators or PARP1 to the regulatory region 5′ of *Shh* rely on catalytically active PARP1.

DISCUSSION

A popular model of enhancer-promoter communication has involved chromatin looping to juxtapose the two elements in 3D nuclear space (Vernimmen and Bickmore, 2015), and we have provided visual evidence that supports this model in the context of long-range gene activation of *Shh* by the ZRS enhancer during limb development (Williamson et al., 2016). However, recent live-cell imaging approaches challenge the idea of stable enhancer-promoter loops as the basis for all enhancer-promoter communication (Fukaya et al., 2016; Alexander et al., 2019).

Here, we have analyzed the spatial relation between *Shh* and its neural enhancers SBE6, SBE4, and SBE2/3 using FISH and 5C during neural differentiation and *in vivo*. We found evidence for a decreased rather than an increased frequency of enhancer-promoter juxtaposition in *Shh*-expressing cells and enhancer-promoter distances in the regulatory domain upstream of *Shh* increase (Figures 1 and 2). We recapitulated this using synthetic activators (based upon TALE-mediated recruitment of VP16) to either induce *Shh* expression directly through activator recruitment to the promoter or induce expression from a distance through recruitment to distal enhancers up to 400 kb away in the *Shh* regulatory domain. Activation from a distance recapitulated the increased enhancer-promoter separation and the decreased enhancer-promoter co-localization that we saw during NPC differentiation (Figure 3). Similar results were seen by the distal recruitment of an endogenous activator (Six3) or a co-activator (Mediator) (Figure 4). There is no evidence for bimodality in our datasets that may suggest a small proportion of looped alleles, although we cannot exclude that such structures are extremely transient.

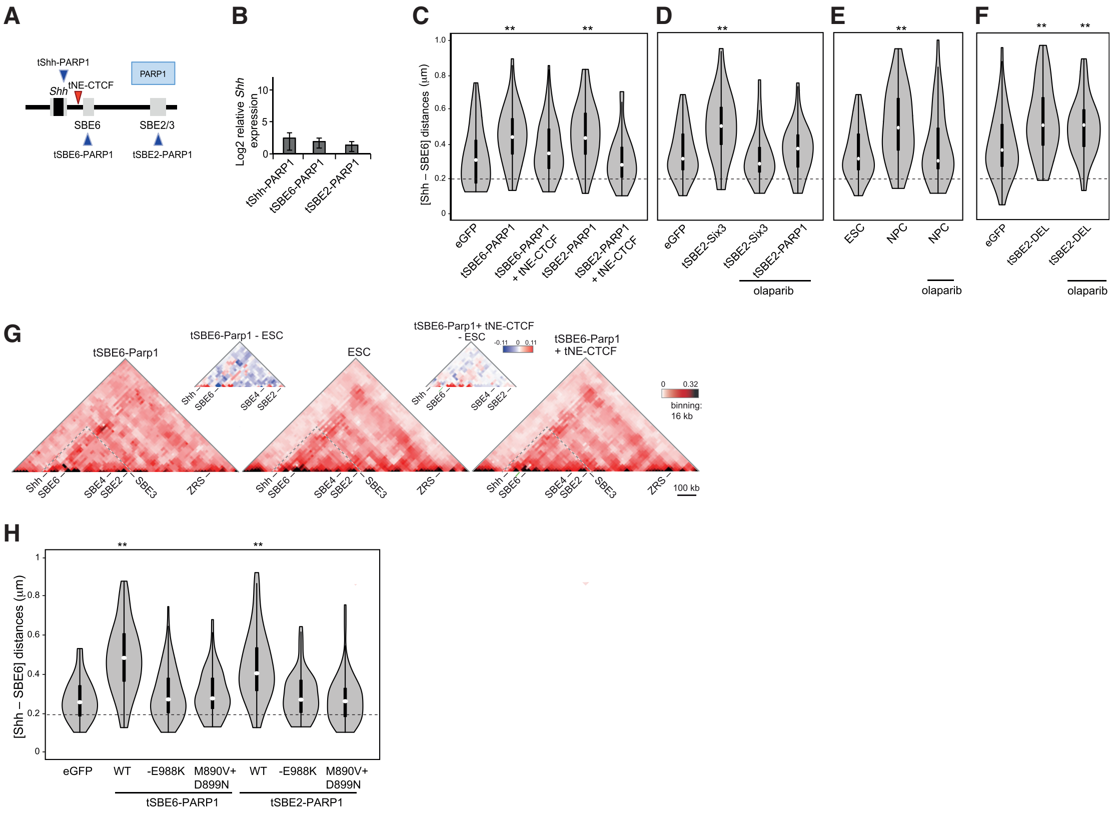

Figure 6. PARP1 Catalytic Activity Decreases Enhancer-Promoter Proximity

(A) Schematic of TALEs that target PARP1 to the *Shh* promoter (tShh), SBE6, or SBE2.

(B) Log2 mRNA levels of *Shh* relative to *Gapdh* in ESCs expressing the TALE-PARP1 constructs shown in (A). Data show means (±SEMs) of five biological replicates normalized to ESCs expressing eGFP.

(C) Violin plots representing one biological replicate of Shh-SBE6 inter-probe distances (μm) in ESCs expressing eGFP, tSBE6-PARP1, tSBE6-PARP1+tNE-CTCF, tSBE2-PARP1, and tSBE2-PARP1+tNE-C*TCF.* **p < 0.01.

(D) As in (C) but for ESCs expressing eGFP and tSBE2-Six3, and then in tSBE2-Six3 or tSBE2-PARP1-expressing cells treated with olaparib.

(E) Violin plots representing one biological replicate of Shh-SBE6 inter-probe distances in ESCs or NPCs and in NPCs treated with olaparib.

(F) As in (D), but for ESCs expressing eGFP or tShh-DEL with and without olaparib treatment. **p < 0.01. Statistical data and replicate experiments are shown in Table S6.

(G) 5C heatmaps of the *Shh* regulatory region (chr5:28604000-29780000) with 16 kb binning and smoothing for ESCs and for ESCs expressing TALE-PARP1 fusions targeting SBE6 or ESCs co-expressing tSBE6-PARP1 and TALE-CTCF targeting NE. Difference 5C plots are shown above the main 5C plots. Data for a replicate experiment are shown in Figure S5.

(H) Violin plots representing one biological replicate of Shh-SBE6 inter-probe distances (μm) in ESCs expressing tSBE6-PARP1 or tSBE2-PARP1, PARP1 catalytic mutant E988K, or PARP1 catalytic double mutant M890V+D899N. **p < 0.01.

Statistical data relating to FISH data are shown in Table S7.

CTCF bound at cognate binding sites is known to alter enhancer and promoter communication (Ali et al., 2016) and could be consistent with our observation that TALE-mediated CTCF recruitment blocks the increase in enhancer-Shh separation induced by activator and co-activator recruitment to enhancers (Figure 5). Overall, our data do not seem compatible with enhancer-promoter loops being essential for enhancer-driven gene transcription.

PARP and Enhancer Activation

We could also induce enhancer-promoter separation at *Shh* by recruiting catalytically active PARP1 and could block it with a PARP inhibitor (olaparib) (Figure 6). Although PARP1 is usually studied in the context of DNA damage sensing and repair, it has also been associated with the regulation of gene expression and with regulatory regions (Nalabothula et al., 2015). PARP and Parp1-dependent PARylation have been implicated in gene regulation from distal enhancer elements that are controlled by nuclear hormone receptors (Sawatsubashi et al., 2004; Schiewer et al., 2012; Wright et al., 2012; Zhang et al., 2013). PARylation is also known to modify the insulating and enhancer blocking activity of CTCF (Yu et al., 2004).

PARylation of histones leads to the decompaction of nucleosome arrays *in vitro* (Huletsky et al., 1989; Poirier et al., 1982) and *in vivo* (Petesch and Lis, 2012; Tulin and Spradling, 2003) and may facilitate chromatin access for factors involved in transcriptional activation, or it may increase the mobility and nuclear search space for enhancers (Gu et al., 2018). The

nucleic acid-like PAR chains can also seed phase separation through liquid de-mixing (Altmeyer et al., 2015), and PARylation has been proposed to be involved in the formation of dynamic non-membrane-bound subnuclear and subcellular compartments involved in a variety of processes, including DNA damage and post-transcriptional regulation (Leung et al., 2011).

Enhancer Condensates and Chromatin Organization

Enhancer function has recently been reconsidered from the perspective of liquid-liquid phase separation (LLPS). It has been proposed that TFs bound at enhancers drive LLPS, nucleating a high concentration of activators, co-activators, and the transcriptional machinery into dynamic condensates to drive transcription (Hnisz et al., 2017). Target genes of super-enhancers can be shown to co-localize at some frequency with condensates that appear to be on the order of several hundreds of nanometers in diameter (Cho et al., 2018; Chong et al., 2018; Sabari et al., 2018; Boija et al., 2018). However, the spatial relation between enhancers and their target genes has not been studied in this context. A recent study indicates that such condensates can physically affect chromatin structure, pushing chromatin out, and in turn, a more open flexible chromatin can facilitate LLPS and condensate formation (Shin et al., 2018). This could be consistent with the increased intra-nuclear distances we have seen between enhancers and their target promoters. Future work should explore the topology of the chromatin linking distal enhancers to their target gene promoters in relation to condensates of the activators and co-activators that are nucleated by these enhancers.

STAR★METHODS

Detailed methods are provided in the online version of this paper and include the following:

- KEY RESOURCES TABLE
- LEAD CONTACT AND MATERIALS AVAILABILITY
- EXPERIMENTAL MODEL AND SUBJECT DETAILS
- METHOD DETAILS
  - Cell Culture, differentiation and transfection
  - 3D-FISH
  - RNA FISH
  - Image Capture
  - RNA extraction and Real Time quantitative Polymerase Chain Reaction (qRT-PCR)
  - Single Cell RT-qPCR
  - TALE Design & Assembly
  - Chromatin Immunoprecipitation and Microarrays
  - 5C primer, 5C library design and preparation
  - 4SU-seq
- QUANTIFICATION AND STATISTICAL ANALYSIS
  - FISH image analysis
  - qRT-PCR
  - Microarray analysis
  - 5C analysis
  - 4SU-seq analysis
- DATA AND CODE AVAILABILITY

SUPPLEMENTAL INFORMATION

Supplemental Information can be found online at https://doi.org/10.1016/j.molcel.2019.07.038.

ACKNOWLEDGMENTS

We thank the staff of the Institute of Genetics and Molecular Medicine (IGMM) fluorescence-activated cell sorting (FACS) and imaging facilities for their invaluable technical help. This work was supported by a Ph.D. studentship to N.S.B., funded through a grant from the UK Medical Research Council to the Edinburgh Super Resolution Imaging Consortium (ESRIC). Work in the group of W.A.B. is supported by MRC University Unit grant MC_UU_00007/2.

AUTHOR CONTRIBUTIONS

N.S.B. conducted most of the experiments and data analysis, prepared the figures, and wrote the manuscript. I.W. assisted with the 5C experiments and manuscript editing. L.K. performed the RNA FISH. R.S.I. assisted with 4sU sequencing (4sU-seq), ChIP, and microarray analyses and manuscript editing. S.B. assisted with tissue section FISH. D.S. helped with ESC to NPC differentiation. G.R.G. assisted with the microarray data processing and statistical analyses. P.T. assisted with TALE design and building. W.A.B. conceived of the study, designed the study, coordinated the study, and wrote the manuscript. All of the authors gave final approval for publication.

DECLARATION OF INTERESTS

The authors declare no competing interests.

Received: June 21, 2018
Revised: May 8, 2019
Accepted: July 29, 2019
Published: September 4, 2019

REFERENCES

Alexander, J.M., Guan, J., Li, B., Maliskova, L., Song, M., Shen, Y., Huang, B., Lomvardas, S., and Weiner, O.D. (2019). Live-cell imaging reveals enhancer-dependent *Sox2* transcription in the absence of enhancer proximity. eLife *8*, e41769.

Ali, T., Renkawitz, R., and Bartkuhn, M. (2016). Insulators and domains of gene expression. Curr. Opin. Genet. Dev. *37*, 17–26.

Altmeyer, M., Neelsen, K.J., Teloni, F., Pozdnyakova, I., Pellegrino, S., Grøfte, M., Rask, M.D., Streicher, W., Jungmichel, S., Nielsen, M.L., and Lukas, J. (2015). Liquid demixing of intrinsically disordered proteins is seeded by poly(ADP-ribose). Nat. Commun. *6*, 8088.

Anderson, E., and Hill, R.E. (2014). Long range regulation of the sonic hedgehog gene. Curr. Opin. Genet. Dev. *27*, 54–59.

Bartman, C.R., Hsu, S.C., Hsiung, C.C.-S., Raj, A., and Blobel, G.A. (2016). Enhancer Regulation of Transcriptional Bursting Parameters Revealed by Forced Chromatin Looping. Mol. Cell *62*, 237–247.

Benabdallah, N.S., and Bickmore, W.A. (2015). Regulatory Domains and Their Mechanisms. Cold Spring Harb. Symp. Quant. Biol. *80*, 45–51.

Benabdallah, N.S., Gautier, P., Hekimoglu-Balkan, B., Lettice, L.A., Bhatia, S., and Bickmore, W.A. (2016). SBE6: a novel long-range enhancer involved in driving sonic hedgehog expression in neural progenitor cells. Open Biol. *6*, 160197.

Berlivet, S., Paquette, D., Dumouchel, A., Langlais, D., Dostie, J., and Kmita, M. (2013). Clustering of tissue-specific sub-TADs accompanies the regulation of HoxA genes in developing limbs. PLoS Genet. *9*, e1004018.

Boija, A., Klein, I.A., Sabari, B.R., Dall'Agnese, A., Coffey, E.L., Zamudio, A.V., Li, C.H., Shrinivas, K., Manteiga, J.C., Hannett, N.M., et al. (2018). Transcription Factors Activate Genes through the Phase-Separation Capacity of Their Activation Domains. Cell *175*, 1842–1855.e16.

Brown, J.M., Roberts, N.A., Graham, B., Waithe, D., Lagerholm, C., Telenius, J.M., De Ornellas, S., Oudelaar, A.M., Scott, C., Szczerbal, I., et al. (2018). A tissue-specific self-interacting chromatin domain forms independently of enhancer-promoter interactions. Nat. Commun. *9*, 3849.

Bulger, M., and Groudine, M. (2011). Functional and mechanistic diversity of distal transcription enhancers. Cell *144*, 327–339.

Burgess-Beusse, B., Farrell, C., Gaszner, M., Litt, M., Mutskov, V., Recillas-Targa, F., Simpson, M., West, A., and Felsenfeld, G. (2002). The insulation of genes from external enhancers and silencing chromatin. Proc. Natl. Acad. Sci. USA *99* (*Suppl 4*), 16433–16437.

Carpenter, A.E., Memedula, S., Plutz, M.J., and Belmont, A.S. (2005). Common effects of acidic activators on large-scale chromatin structure and transcription. Mol. Cell. Biol. *25*, 958–968.

Carter, D., Chakalova, L., Osborne, C.S., Dai, Y.F., and Fraser, P. (2002). Long-range chromatin regulatory interactions in vivo. Nat. Genet. *32*, 623–626.

Cho, W.K., Spille, J.H., Hecht, M., Lee, C., Li, C., Grube, V., and Cisse, I.I. (2018). Mediator and RNA polymerase II clusters associate in transcription-dependent condensates. Science *361*, 412–415.

Chong, S., Dugast-Darzacq, C., Liu, Z., Dong, P., Dailey, G.M., Cattoglio, C., Heckert, A., Banala, S., Lavis, L., Darzacq, X., and Tjian, R. (2018). Imaging dynamic and selective low-complexity domain interactions that control gene transcription. Science *361*, eaar2555.

Dalerba, P., Kalisky, T., Sahoo, D., Rajendran, P.S., Rothenberg, M.E., Leyrat, A.A., Sim, S., Okamoto, J., Johnston, D.M., Qian, D., et al. (2011). Single-cell dissection of transcriptional heterogeneity in human colon tumors. Nat. Biotechnol. *29*, 1120–1127.

Deng, W., Lee, J., Wang, H., Miller, J., Reik, A., Gregory, P.D., Dean, A., and Blobel, G.A. (2012). Controlling long-range genomic interactions at a native locus by targeted tethering of a looping factor. Cell *149*, 1233–1244.

Deng, W., Rupon, J.W., Krivega, I., Breda, L., Motta, I., Jahn, K.S., Reik, A., Gregory, P.D., Rivella, S., Dean, A., and Blobel, G.A. (2014). Reactivation of developmentally silenced globin genes by forced chromatin looping. Cell *158*, 849–860.

Ding, Q., Lee, Y.-K., Schaefer, E.A.K., Peters, D.T., Veres, A., Kim, K., Kuperwasser, N., Motola, D.L., Meissner, T.B., Hendriks, W.T., et al. (2013). A TALEN genome-editing system for generating human stem cell-based disease models. Cell Stem Cell *12*, 238–251.

Doyle, E.L., Booher, N.J., Standage, D.S., Voytas, D.F., Brendel, V.P., Vandyk, J.K., and Bogdanove, A.J. (2012). TAL Effector-Nucleotide Targeter (TALE-NT) 2.0: tools for TAL effector design and target prediction. Nucleic Acids Res. *40*, W117–W122.

Engel, N., Raval, A.K., Thorvaldsen, J.L., and Bartolomei, S.M. (2008). Three-dimensional conformation at the H19/Igf2 locus supports a model of enhancer tracking. Hum. Mol. Genet. *17*, 3021–3029.

Eskeland, R., Leeb, M., Grimes, G.R., Kress, C., Boyle, S., Sproul, D., Gilbert, N., Fan, Y., Skoultchi, A.I., Wutz, A., and Bickmore, W.A. (2010). Ring1B compacts chromatin structure and represses gene expression independent of histone ubiquitination. Mol. Cell *38*, 452–464.

Fraser, J., Ethier, S.D., Miura, H., and Dostie, J. (2012). A Torrent of data: mapping chromatin organization using 5C and high-throughput sequencing. Methods Enzymol. *513*, 113–141.

Fukaya, T., Lim, B., and Levine, M. (2016). Enhancer Control of Transcriptional Bursting. Cell *166*, 358–368.

Geng, X., Speirs, C., Lagutin, O., Inbal, A., Liu, W., Solnica-Krezel, L., Jeong, Y., Epstein, D.J., and Oliver, G. (2008). Haploinsufficiency of Six3 fails to activate Sonic hedgehog expression in the ventral forebrain and causes holoprosencephaly. Dev. Cell *15*, 236–247.

Gilbert, N., Boyle, S., Sutherland, H., de Las Heras, J., Allan, J., Jenuwein, T., and Bickmore, W.A. (2003). Formation of facultative heterochromatin in the absence of HP1. EMBO J. *22*, 5540–5550.

Gu, B., Swigut, T., Spencley, A., Bauer, M.R., Chung, M., Meyer, T., and Wysocka, J. (2018). Transcription-coupled changes in nuclear mobility of mammalian cis-regulatory elements. Science *359*, 1050–1055.

Hnisz, D., Shrinivas, K., Young, R.A., Chakraborty, A.K., and Sharp, P.A. (2017). A Phase Separation Model for Transcriptional Control. Cell *169*, 13–23.

Huletsky, A., de Murcia, G., Muller, S., Hengartner, M., Ménard, L., Lamarre, D., and Poirier, G.G. (1989). The effect of poly(ADP-ribosyl)ation on native and H1-depleted chromatin. A role of poly(ADP-ribosyl)ation on core nucleosome structure. J. Biol. Chem. *264*, 8878–8886.

Illingworth, R.S., Moffat, M., Mann, A.R., Read, D., Hunter, C.J., Pradeepa, M.M., Adams, I.R., and Bickmore, W.A. (2015). The E3 ubiquitin ligase activity of RING1B is not essential for early mouse development. Genes Dev. *29*, 1897–1902.

Jeong, Y., El-Jaick, K., Roessler, E., Muenke, M., and Epstein, D.J. (2006). A functional screen for sonic hedgehog regulatory elements across a 1 Mb interval identifies long-range ventral forebrain enhancers. Development *133*, 761–772.

Jeong, Y., Leskow, F.C., El-Jaick, K., Roessler, E., Muenke, M., Yocum, A., Dubourg, C., Li, X., Geng, X., Oliver, G., and Epstein, D.J. (2008). Regulation of a remote Shh forebrain enhancer by the Six3 homeoprotein. Nat. Genet. *40*, 1348–1353.

Kagey, M.H., Newman, J.J., Bilodeau, S., Zhan, Y., Orlando, D.A., van Berkum, N.L., Ebmeier, C.C., Goossens, J., Rahl, P.B., Levine, S.S., et al. (2010). Mediator and cohesin connect gene expression and chromatin architecture. Nature *467*, 430–435.

Kim, T.-K., Hemberg, M., Gray, J.M., Costa, A.M., Bear, D.M., Wu, J., Harmin, D.A., Laptewicz, M., Barbara-Haley, K., Kuersten, S., et al. (2010). Widespread transcription at neuronal activity-regulated enhancers. Nature *465*, 182–187.

Leung, A.K., Vyas, S., Rood, J.E., Bhutkar, A., Sharp, P.A., and Chang, P. (2011). Poly(ADP-ribose) regulates stress responses and microRNA activity in the cytoplasm. Mol. Cell *42*, 489–499.

Milbradt, A.G., Kulkarni, M., Yi, T., Takeuchi, K., Sun, Z.-Y.J., Luna, R.E., Selenko, P., Näär, A.M., and Wagner, G. (2011). Structure of the VP16 transactivator target in the Mediator. Nat. Struct. Mol. Biol. *18*, 410–415.

Mittler, G., Stühler, T., Santolin, L., Uhlmann, T., Kremmer, E., Lottspeich, F., Berti, L., and Meisterernst, M. (2003). A novel docking site on Mediator is critical for activation by VP16 in mammalian cells. EMBO J. *22*, 6494–6504.

Morey, C., Da Silva, N.R., Perry, P., and Bickmore, W.A. (2007). Nuclear reorganisation and chromatin decondensation are conserved, but distinct, mechanisms linked to Hox gene activation. Development *134*, 909–919.

Morgan, S.L., Mariano, N.C., Bermudez, A., Arruda, N.L., Wu, F., Luo, Y., Shankar, G., Jia, L., Chen, H., Hu, J.F., et al. (2017). Manipulation of nuclear architecture through CRISPR-mediated chromosomal looping. Nat. Commun. *8*, 15993.

Nalabothula, N., Al-jumaily, T., Eteleeb, A.M., Flight, R.M., Xiaorong, S., Moseley, H., Rouchka, E.C., and Fondufe-Mittendorf, Y.N. (2015). Genome-Wide Profiling of PARP1 Reveals an Interplay with Gene Regulatory Regions and DNA Methylation. PLoS One *10*, e0135410.

Naughton, C., Avlonitis, N., Corless, S., Prendergast, J.G., Mati, I.K., Eijk, P.P., Cockroft, S.L., Bradley, M., Ylstra, B., and Gilbert, N. (2013). Transcription forms and remodels supercoiling domains unfolding large-scale chromatin structures. Nat. Struct. Mol. Biol. *20*, 387–395.

Petesch, S.J., and Lis, J.T. (2012). Activator-induced spread of poly(ADP-ribose) polymerase promotes nucleosome loss at Hsp70. Mol. Cell *45*, 64–74.

Phillips-Cremins, J.E., Sauria, M.E., Sanyal, A., Gerasimova, T.I., Lajoie, B.R., Bell, J.S., Ong, C.T., Hookway, T.A., Guo, C., Sun, Y., et al. (2013). Architectural protein subclasses shape 3D organization of genomes during lineage commitment. Cell *153*, 1281–1295.

Poirier, G.G., de Murcia, G., Jongstra-Bilen, J., Niedergang, C., and Mandel, P. (1982). Poly(ADP-ribosyl)ation of polynucleosomes causes relaxation of chromatin structure. Proc. Natl. Acad. Sci. USA *79*, 3423–3427.

Pollard, S.M., Benchoua, A., and Lowell, S. (2006). Neural stem cells, neurons, and glia. Methods Enzymol. *418*, 151–169.

Pradeepa, M.M., Grimes, G.R., Kumar, Y., Olley, G., Taylor, G.C.A., Schneider, R., and Bickmore, W.A. (2016). Histone H3 globular domain acetylation identifies a new class of enhancers. Nat. Genet. *48*, 681–686.

Rabani, M., Levin, J.Z., Fan, L., Adiconis, X., Raychowdhury, R., Garber, M., Gnirke, A., Nusbaum, C., Hacohen, N., Friedman, N., et al. (2011). Metabolic labeling of RNA uncovers principles of RNA production and degradation dynamics in mammalian cells. Nat. Biotechnol. *29*, 436–442.

Rada-Iglesias, A., Bajpai, R., Swigut, T., Brugmann, S.A., Flynn, R.A., and Wysocka, J. (2011). A unique chromatin signature uncovers early developmental enhancers in humans. Nature *470*, 279–283.

Rolli, V., O'Farrell, M., Ménissier-de Murcia, J., and de Murcia, G. (1997). Random mutagenesis of the poly(ADP-ribose) polymerase catalytic domain reveals amino acids involved in polymer branching. Biochemistry *36*, 12147–12154.

Sabari, B.R., Dall'Agnese, A., Boija, A., Klein, I.A., Coffey, E.L., Shrinivas, K., Abraham, B.J., Hannett, N.M., Zamudio, A.V., Manteiga, J.C., et al. (2018). Coactivator condensation at super-enhancers links phase separation and gene control. Science *361*, eaar3958.

Sawatsubashi, S., Maki, A., Ito, S., Shirode, Y., Suzuki, E., Zhao, Y., Yamagata, K., Kouzmenko, A., Takeyama, K., and Kato, S. (2004). Ecdysone receptor-dependent gene regulation mediates histone poly(ADP-ribosyl)ation. Biochem. Biophys. Res. Commun. *320*, 268–272.

Schiewer, M.J., Goodwin, J.F., Han, S., Brenner, J.C., Augello, M.A., Dean, J.L., Liu, F., Planck, J.L., Ravindranathan, P., Chinnaiyan, A.M., et al. (2012). Dual roles of PARP-1 promote cancer growth and progression. Cancer Discov. *2*, 1134–1149.

Shin, Y., Chang, Y.C., Lee, D.S.W., Berry, J., Sanders, D.W., Ronceray, P., Wingreen, N.S., Haataja, M., and Brangwynne, C.P. (2018). Liquid Nuclear Condensates Mechanically Sense and Restructure the Genome. Cell *175*, 1481–1491.e13.

Shlyueva, D., Stampfel, G., and Stark, A. (2014). Transcriptional enhancers: from properties to genome-wide predictions. Nat. Rev. Genet. *15*, 272–286.

Singh, H.R., Nardozza, A.P., Möller, I.R., Knobloch, G., Kistemaker, H.A.V., Hassler, M., Harrer, N., Blessing, C., Eustermann, S., Kotthoff, C., et al. (2017). A Poly-ADP-Ribose Trigger Releases the Auto-Inhibition of a Chromatin Remodeling Oncogene. Mol. Cell *68*, 860–871.e7.

Smallwood, A., and Ren, B. (2013). Genome organization and long-range regulation of gene expression by enhancers. Curr. Opin. Cell Biol. *25*, 387–394.

Soutourina, J. (2018). Transcription regulation by the Mediator complex. Nat. Rev. Mol. Cell Biol. *19*, 262–274.

Taylor, G.C.A., Eskeland, R., Hekimoglu-Balkan, B., Pradeepa, M.M., and Bickmore, W.A. (2013). H4K16 acetylation marks active genes and enhancers of embryonic stem cells, but does not alter chromatin compaction. Genome Res. *23*, 2053–2065.

Therizols, P., Illingworth, R.S., Courilleau, C., Boyle, S., Wood, A.J., and Bickmore, W.A. (2014). Chromatin decondensation is sufficient to alter nuclear organization in embryonic stem cells. Science *346*, 1238–1242.

Tolhuis, B., Palstra, R.J., Splinter, E., Grosveld, F., and de Laat, W. (2002). Looping and interaction between hypersensitive sites in the active beta-globin locus. Mol. Cell *10*, 1453–1465.

Tulin, A., and Spradling, A. (2003). Chromatin loosening by poly(ADP)-ribose polymerase (PARP) at Drosophila puff loci. Science *299*, 560–562.

Tulin, A., Chinenov, Y., and Spradling, A. (2003). Regulation of chromatin structure and gene activity by poly(ADP-ribose) polymerases. Curr. Top. Dev. Biol. *56*, 55–83.

Vernimmen, D., De Gobbi, M., Sloane-Stanley, J.A., Wood, W.G., and Higgs, D.R. (2007). Long-range chromosomal interactions regulate the timing of the transition between poised and active gene expression. EMBO J. *26*, 2041–2051.

Vernimmen, D., and Bickmore, W.A. (2015). The Hierarchy of Transcriptional Activation: From Enhancer to Promoter. Trends Genet. *31*, 696–708.

Vojnic, E., Mourão, A., Seizl, M., Simon, B., Wenzeck, L., Larivière, L., Baumli, S., Baumgart, K., Meisterernst, M., Sattler, M., and Cramer, P. (2011). Structure and VP16 binding of the Mediator Med25 activator interaction domain. Nat. Struct. Mol. Biol. *18*, 404–409.

Williamson, I., Berlivet, S., Eskeland, R., Boyle, S., Illingworth, R.S., Paquette, D., Dostie, J., and Bickmore, W.A. (2014). Spatial genome organization: contrasting views from chromosome conformation capture and fluorescence in situ hybridization. Genes Dev. *28*, 2778–2791.

Williamson, I., Lettice, L.A., Hill, R.E., and Bickmore, W.A. (2016). Shh and ZRS enhancer colocalisation is specific to the zone of polarising activity. Development *143*, 2994–3001.

Wright, R.H.G., Castellano, G., Bonet, J., Le Dily, F., Font-Mateu, J., Ballaré, C., Nacht, A.S., Soronellas, D., Oliva, B., and Beato, M. (2012). CDK2-dependent activation of PARP-1 is required for hormonal gene regulation in breast cancer cells. Genes Dev. *26*, 1972–1983.

Yao, Y., Minor, P.J., Zhao, Y.T., Jeong, Y., Pani, A.M., King, A.N., Symmons, O., Gan, L., Cardoso, W.V., Spitz, F., et al. (2016). Cis-regulatory architecture of a brain signaling center predates the origin of chordates. Nat. Genet. *48*, 575–580.

Ying, Q.-L., Stavridis, M., Griffiths, D., Li, M., and Smith, A. (2003). Conversion of embryonic stem cells into neuroectodermal precursors in adherent monoculture. Nat. Biotechnol. *21*, 183–186.

Yu, W., Ginjala, V., Pant, V., Chernukhin, I., Whitehead, J., Docquier, F., Farrar, D., Tavoosidana, G., Mukhopadhyay, R., Kanduri, C., et al. (2004). Poly(ADP-ribosyl)ation regulates CTCF-dependent chromatin insulation. Nat. Genet. *36*, 1105–1110.

Zhang, F., Cong, L., Lodato, S., Kosuri, S., Church, G.M., and Arlotta, P. (2011). Efficient construction of sequence-specific TAL effectors for modulating mammalian transcription. Nat. Biotechnol. *29*, 149–153.

Zhang, F., Wang, Y., Wang, L., Luo, X., Huang, K., Wang, C., Du, M., Liu, F., Luo, T., Huang, D., and Huang, K. (2013). Poly(ADP-ribose) polymerase 1 is a key regulator of estrogen receptor α-dependent gene transcription. J. Biol. Chem. *288*, 11348–11357.

Zhao, H., and Dean, A. (2004). An insulator blocks spreading of histone acetylation and interferes with RNA polymerase II transfer between an enhancer and gene. Nucleic Acids Res. *32*, 4903–4919.

Zhu, X., Ling, J., Zhang, L., Pi, W., Wu, M., and Tuan, D. (2007). A facilitated tracking and transcription mechanism of long-range enhancer function. Nucleic Acids Res. *35*, 5532–5544.

STAR★METHODS

KEY RESOURCES TABLE

REAGENT or RESOURCE	SOURCE	IDENTIFIER
Antibodies		
MED12	Bethyl laboratories	Cat# A300-774A; RRID:AB_669756
H3K27ac	Millipore	Cat# 07-360; RRID:AB_310550
Bacterial and Virus Strains		
Modified TALEN backbone Vp64	Therizols et al. 2014	N/A
Fosmid FISH probe	BACPAC resource	See Table S8
Chemicals, Peptides, and Recombinant Proteins		
Protease Inhibitor Cocktail Set III, EDTA-Free	Calbiochem, Merck	539134-1SET
PhosSTOP	Roche, Merck	4906837001
GMEM	Gibco, ThermoFisher	11710-035
DMEM/F-12, HEPES	Gibco, ThermoFisher	11330-032
Neurobasal Medium	Gibco, ThermoFisher	21103-049
B-27 Supplement (50X), serum free	Gibco, ThermoFisher	17504044
N-2 Supplement (100X)	Gibco, ThermoFisher	17502048
2-Mercaptoethanol	ThermoFisher	31350-010
Lipofectamine 2000 Transfection Reagent	Invitrogen, ThermoFisher	11668027
Olaparib (AZD2281, Ku-0059436)	Selleck Chemicals	S1060
Green dUTP	Abbott Molecular	02N32-050, 00884999002913
ChromaTide Alexa Fluor 594-5-dUTP	Invitrogen, ThermoFisher	C11400
Mouse Cot-1 DNA	Invitrogen, ThermoFisher	18440-016
EGS (ethylene glycol bis(succinimidyl succinate)	Thermo Scientific, ThermoFisher	21565
Pierce 16% Formaldehyde (w/v), Methanol-free	Thermo Scientific, ThermoFisher	28908
Proteinase K	Genaxxon bioscience	M3037.0001
4-Thiouridine	Sigma, Merck	T4509
TRIzol Reagent	Invitrogen, ThermoFisher	15596026
EZ-Link HPDP-Biotin	Thermo Scientific, ThermoFisher	21341
SYBR Green I nucleic acid gel stain	Sigma, Merck	S9430
Critical Commercial Assays		
Wizard SV Gel and PCR Clean-Up	Promega	A9282
RNeasy Mini Kit	Qiagen	74106
SuperScript II Reverse Transcriptase	Invitrogen, ThermoFisher	18064014
LightCycler 480 SYBR Green I Master	Roche Diagnostics	507203180
CellsDirect One-Step qRT-PCR Kit	Invitrogen, ThermoFisher	11753500
Musunuru/Cowan Lab TALEN Kit	Addgene	1000000034
Protein G Sepharose, GE Healthcare	Sigma, Merck	GE17-0618-01
GenomePlex Complete Whole Genome Amplification (WGA) Kit	Sigma, Merck	WGA2-50RXN
MinElute PCR Purification Kit	Qiagen	28004
μMACS Streptavidin Kit	Miltenyi	130-074-101
TURBO DNA-free Kit	Invitrogen, ThermoFisher	AM1907M
RNeasy MinElute Cleanup Kit	Qiagen	74204
Low Input RiboMinus Eukaryote System v2	Invitrogen, ThermoFisher	A15027
NEBNext Ultra II Directional RNA Library Prep Kit for Illumina	NEB	E7760

(*Continued on next page*)

Continued

REAGENT or RESOURCE	SOURCE	IDENTIFIER
Deposited Data		
5C sequencing	This paper	GEO: GSE89388
Chip-chip Data Nimblegen 720K, H3K27ac	This paper	GEO: GSE115642
4SU-seq	This paper	GEO: GSE115774
Experimental Models: Cell Lines		
46c mESCs derived from E14tg2A containing Sox1-GFP	Ying et al., 2003	N/A
Experimental Models: Organisms/Strains		
CD1 mice	Williamson et al., 2016	N/A
Oligonucleotides		
RT-PCR Primers	This paper	See Table S9
ChIP-qPCR Primers	This paper	See Table S9
SA domain of LDB1, gBlocks Gene Fragments	IDT	N/A
VWA domain of Med25, gBlocks Gene Fragments	IDT	N/A
CTCF, Invitrogen GeneArt Gene Synthesis	ThermoFisher	N/A
SIX3, Invitrogen GeneArt Gene Synthesis	ThermoFisher	N/A
PARP1, Invitrogen GeneArt Gene Synthesis	ThermoFisher	N/A
PARP1 E988K, Invitrogen GeneArt Gene Synthesis	ThermoFisher	N/A
PARP1 M890V+D899N, Invitrogen GeneArt Gene Synthesis	ThermoFisher	N/A
DELQPASIDP peptide	Carpenter et al., 2005; Therizols et al., 2014	N/A
A-key and P1-key primers (5C library)	Fraser et al., 2012	N/A
5C primers covering USP22 and Shh region	Williamson et al. 2016	N/A
NEBNext Multiplex Oligos for Illumina (Index Primers Set 1)	NEB	E7335
Shh Custom Stellaris RNA FISH Probes	Stellaris RNA FISH	This study
Software and Algorithms		
Nikon Nis-Elements Imaging software	Nikon UK Ltd	https://www.microscope.healthcare.nikon.com/en_EU/products/software/nis-elements
Volocity 3D Image Analysis Softwar	PerkinElmer Inc	https://www.perkinelmer.co.uk/lab-products-and-services/cellular-imaging/volocity-3d-visualization.html
TAL Effector Nucleotide Targeter 2.0 (TALE design)	Doyle et al., 2012	https://tale-nt.cac.cornell.edu/
LightCycler 480 Software	Roche	04898915001
Linear Models for Microarray Data (Limma) Bioconductor package (R)	Bioconductor	http://www.bioconductor.org/packages/devel/bioc//manuals/limma/man/limma.pdf
Torrent 5C data transformation pipeline on Galaxy	Galaxy	https://main.g2.bx.psu.edu
HOMER v4.8 (RNA seq)		http://homer.ucsd.edu/homer/
Other		
H3K27me3 Chip-Seq data	Illingworth et al., 2015	GEO: GSE69955

LEAD CONTACT AND MATERIALS AVAILABILITY

Materials Availability Statement: Further information and requests for resources and reagents should be directed to and will be fulfilled by the Lead Contact, Wendy Bickmore (Wendy.Bickmore@igmm.ed.ac.uk).

EXPERIMENTAL MODEL AND SUBJECT DETAILS

46c male mouse embryonic stem cells (mESCs), derived from E14tg2A, contain a GFP insertion into the Sox1 locus (Ying et al., 2003) were used for this study. mESCs were cultured in GMEM supplemented with 10% fetal bovine serum (FBS), 1000 units/ml LIF, nonessential amino acids, sodium pyruvate, 2-β-mercaptoethanol, L-glutamine and penicillin/streptomycin.

METHOD DETAILS

Cell Culture, differentiation and transfection

ESCs were differentiated into NPCs essentially as described previously (Pollard et al., 2006; Ying et al., 2003).To ensure a homogeneous starting population of ESCs, cells were harvested using trypsin 24 h prior to initiating differentiation and seeded at $3x10^6$ on 0.1% gelatin coated Corning flasks. Differentiation media (1:1 DMEM/F12:Neurobasal medium (GIBCO, #31330-032 and 21103-049 respectively) supplemented with 0.5x B27 (Invitrogen, #17504044), 0.5x N2 (Invitrogen, # 17502048), L-Glutamine and 50 μM 2-β-mercaptoethanol (GIBCO, #31350-010)) was prepared fresh on the day of differentiation. On D0, ESCs were harvested, washed twice with PBS and twice with differentiation media then seeded at a density of $1x10^6$ ESCs on 0.1% gelatin coated T75 Corning flasks (gelatin coated at RT for 3-6 h and washed twice with PBS prior to seeding). Cells were then cultured for 7 days with daily media changes after day 2. NPC differentiation was monitored visually through expression of the GFP reporter.

ESCs were transfected with TALE plasmids using Lipofectamine® 2000 Reagent (Invitrogen, #11668) and FAC-sorted for GFP as previously described (Therizols et al., 2014). Briefly, $1x10^6$ ESCs were transfected in a 6-well plate with 2.5μg of plasmid and 7μl of Lipofectamine. The culture medium was changed 6h after transfection. Transfected cells were FACS sorted based on eGFP expression 24h after transfection and re-seeded on slides or 6-well-plates. Flow cytometric analysis was performed using the 488nm laser of a BD FACSAriaII SORP (Becton Dickinson) with 525/50 nm bandpass filters. BD FACSDiva software (Becton Dickinson, Version 6.1.2) was used for instrument control and data analysis.

For PARP inhibition, 24h after transfection or 5h after FACs olaparib was added to media to a concentration of 10μM for 1.5 h before cells were fixed for FISH.

3D-FISH

$1x10^6$ ESCs or NPCs were seeded on Sigma Poly-Prep glass slides for 5h. Cells were fixed in 4% paraformaldehyde (pFA) for 10 mins at room temperature (r.t.) and then permeabilized using 0.5% TritonX for 10 mins (Eskeland et al., 2010). Fosmid clones were labeled with green-dUTP (Abbott Molecular 02N32-050, 00884999002913) or red-dUTP (ChromaTide Alexa Fluor 594-5-dUTP C11400). Approximately 150 ng of labeled fosmid probes were used per slide, together with 15 μg of mouse Cot1 DNA (GIBCO BRL) and 10 μg salmon sperm DNA. Probes were denatured at 70°C for 5 min, reannealed with CotI DNA for 15 min at 37°C and hybridized to the denatured slides overnight. Slides were incubated in 2XSSC with RNase for 1h at 37°C, dried in a series of EtOH washes and pre-warmed in an oven at 75°C for 15 mins before being denatured at 80°C for 20 mins in 70% formamide/2 × SSC pH 7.5 followed by 3 minutes in ice-cold 70% ethanol, dried in EtOH gradient and hybridized at 37°C overnight. Slides were then washed in 2XSSC at 37°C then in 0.5XSSC at 65°C and finally in 4XSSC-Tween. FISH probes are described in Table S8.

For tissues sections 3D-FISH mouse embryos were collected, fixed, embedded, sectioned and processed as previously described (Morey et al., 2007). Briefly, embryos from CD1 mice were collected at E10.5, fixed in 4% pFa overnight at 4°C, dehydrated through an ethanol series, cleared in xylene and embedded in paraffin blocks. Sections laid on Superfrost slides were heated to 60°C for 20 mins and washed in xylene before rehydration through an ethanol series. Slides were microwaved for 20 mins in 0.1 M citrate pH 6.0 buffer, washed in water and rinsed once in 2 × SSC before use. FISH was performed as described above, but with a denaturation step of 3 minutes at 75°C.

RNA FISH

Custom Stellaris® RNA FISH Probes were designed against Shh nascent mRNA (pool of 48 unique 22-mer probes) by utilizing the Stellaris® RNA FISH Probe Designer (Biosearch Technologies, Inc., Petaluma, CA) (https://www.biosearchtech.com/support/tools/design-software/stellaris-probe-designer, version 4.2). Slides were hybridized with the Shh Stellaris FISH Probe set labeled with Quasar 570 (Biosearch Technologies, Inc.), following the manufacturer's instructions (https://www.biosearchtech.com/support/resources/stellaris-protocols). Briefly, FFPE tissue sections from E10.5 embryos were deparaffinised in xylene, hydrated in ethanol and permeabilised in 70% ethanol overnight at 4°C. Slides were incubated in 10 μg/mL proteinase K in 1X PBS for 20 minutes at 37°C followed washes in 1X PBS and Stellaris Wash Buffer A (#SMF-WA1-60). Shh RNA FISH probes were diluted in Stellaris RNA FISH hybridization buffer (#SMF-HB1-10) to 125 nM and hybridized to slides overnight in humidified chamber at 37°C. Slides were washed 2 × 30 minutes in Wash Buffer A at 37°C, counterstained with 5 ng/mL DAPI, washed in Stellaris Wash Buffer B (#SMF-WB1-20) and mounted in Vectashield.

Image Capture

Super-resolution images from 3D FISH were acquired using structured illumination microscopy (SIM). 3D-SIM images were acquired on a N-SIM (Nikon Instruments, UK) using a 100x Nikon Plan Apo TIRF objective (NA 1.49, oil immersion) and refractive index

matched immersion oil (Nikon Instruments). Images were captured using an Andor DU-897X-5254 EMCCD camera using 405, 488, 561 and 640nm laser lines. Step size for *z* stacks was set to 0.12 μm as required by the manufacturer's software. For each focal plane, 15 images (5 phases, 3 angles) were captured with the NIS-Elements software.

RNA FISH slides were imaged using a Photometrics Coolsnap HQ2 CCD camera and a Zeiss AxioImager A1 fluorescence microscope with a Plan Apochromat 100x 1.4NA objective, a Nikon Intensilight Mercury based light source (Nikon UK Ltd, Kingston-on-Thames, UK) and Chroma #89014ET (3 color) single excitation and emission filters (Chroma Technology Corp., Rockingham, VT) with the excitation and emission filters installed in Prior motorised filter wheels. A piezoelectrically driven objective mount (PIFOC model P-721, Physik Instrumente GmbH & Co, Karlsruhe) was used to control movement in the z dimension. Step size for z stacks was set to 0.2 μm. Hardware control and image capture were performed using Nikon Nis-Elements software (Nikon UK Ltd, Kingston-on-Thames, UK). Images were deconvolved using a calculated PSF in Volocity (PerkinElmer Inc, Waltham MA). Image analysis was carried out using the Quantitation module of Volocity (PerkinElmer Inc, Waltham MA).

RNA extraction and Real Time quantitative Polymerase Chain Reaction (qRT-PCR)

RNA was prepared using RNeasy mini kit (QIAGEN) according to the manufacturer's protocol, including a DNaseI (QIAGEN) treatment for 15 mins at r.t. cDNA was synthesized from 2 μg purified RNA with Superscript II reverse transcriptase (Invitrogen) primed with random hexamers (Promega).

Real-time PCR was carried on the Roche LightCycler 480 Real-Time PCR System using a Lightcycler 480 Sybr Green detection kit (Roche). The real-time thermal cycler was programmed as follows: 15 min Hotstart; 44 PCR cycles (95°C for 15 s, 55°C for 30 s, 72°C for 30 s). A standard curve for each primer set was obtained using a mix of each of the cDNAs. Primers are listed in Table S9. *Ptn* and *Nrp1* primers were taken from (Therizols et al., 2014).

Single Cell RT-qPCR

RNA reverse transcription and cDNA pre-amplification from single cells were performed as previously described (Dalerba et al., 2011) with some modifications. Each well of a 96-well PCR plate was loaded with 5 μl 2x Reaction Mix, 0.2 μl Superscript III RT/Platinum Taq Mix with RNaseOUT Ribonuclease Inhibitor (Invitrogen Cells Direct One-Step qRT-PCR kit, Life Technologies), 2.5 μl primer mix (containing 200 nM of each gene-specific primer), 1.3 μl H_2O. Single-cell suspensions were sorted on their GFP reporter into separate wells of the 96-well PCR plate. 32 cells were sorted into one well, to be used for serial dilution for generation of qRT-PCR standard curves. RNA reverse transcription and 22 cycles of cDNA pre-amplification were performed as previously described (Dalerba et al., 2011). The cDNA was diluted 1:5 in H_2O. For the qRT-PCR, 9 μl of cDNA was loaded into a 96-well plate with one 96-well plate loaded with one primer pair i.e *Gapdh* or *Shh*.

TALE Design & Assembly

TALEs were designed using TAL Effector Nucleotide Targeter 2.0 software (Doyle et al., 2012) and the assembly was performed following the protocol described in (Therizols et al., 2014). TALE DNA binding domains specific for 16 base pairs of the *Shh* promoter, SBE6, SBE2, and NE were assembled following the methods described in (Ding et al., 2013). Four pre-assembled multimeric TALE repeat modules (three 4-mer and one 3-mer) were assembled into a modified TALEN backbone in which the BamHI-BsrGI fragment containing hFokI2-2A-eGFP was replaced by a gBlocks® (IDT) fragment encoding Vp64-2A-eGFP (Therizols et al., 2014). The BamHI-BglII fragment containing Vp64 of the TALE-Vp64 plasmid was deleted to generate Tale-Δ.

To generate other TALE-fusions, the BamHI-NheI cloning site was further used to fuse the TALE with; the SA domain of LDB1 designed and ordered from gBlocks® (IDT), with CTCF, PARP1 and PARP1 mutants (PARP1 E988K and M890V+D899N) designed and ordered from GeneArt® (Life Technologies). To generate Tale-BP, the BamHI-NheI fragment containing Vp64 was replaced by a double strand oligonucleotide encoding the DELQPASIDP peptide (Carpenter et al., 2005).

Chromatin Immunoprecipitation and Microarrays

For cross-link ChIP, 0.5-1x10^7 ESC were first cross-linked with 2 mM EGS (Pearce, Thermo Scientific, #21565) in PBS for 60 min at r.t. Methanol-free formaldehyde was then added at a final concentration of 1% (Thermo Scientific Pierce, PN28906) for 10 min at r.t. and quenched for 5 min with 125mM glycine and then washing in PBS. All buffers were supplemented with the following additives just prior to use: 0.2 mM PMSF, 1 mM DTT, 1x Protease inhibitors (Calbiochem, 539134-1SET) and 1x phosphatase inhibitors (Roche, PhosSTOP, 04906837001). Purified DNA was isolated using a QIAquick PCR Purification Kit (QIAGEN). Med12 ChIP was performed as previously described (Vernimmen et al., 2007) using Med12 antibody (Bethyl laboratories, A300-774A). Quantitative (q)PCR was performed on a LightCycler480 (Roche) using the same guideline as for qRT-PCR. ChIP qPCR primers are displayed in Table S9.

For examination of histone H3 K27 acetylation (H3K27ac) by native ChIP, nuclei were prepared and resuspended in NB-R as previously described (Gilbert et al., 2003). Nuclei corresponding to 0.5-1x10^7 ESCs were digested with 50-80 Boehringer units of MNase (Sigma) for 10 min at r.t. in the presence of 20 μg RNase A to obtain a chromatin ladder enriched in tri-, tetra-, and some pentanucleosomes. The reaction was stopped by adding equal volume of Stop Buffer (215 mM NaCl, 10 mM TrisHCl pH 8, 20 mM EDTA, 5.5% Sucrose, 2% Triton X-100, 0.2 mM PMSF 1 mM DTT and complete protease inhibitor cocktail) and incubated on ice overnight. Between 50-150 μg released chromatin were pre-cleared with Protein G Sepharose (GE Healthcare) for 2 hr and mixed with 10 μg

prebound H3K27ac antibody (Millipore 07-360) in the presence of 100 μg BSA and incubated for 3 hr at 4°C. Beads were then washed 3x with Wash Buffer (150 mM NaCl, 10 mM TrisHCl pH 8, 2 mM EDTA, 1% NP40, 1% Sodium deoxycholate, 0.2mM PMSF, 1mM DTT and protease inhibitor cocktail) and once in TE. Bound complexes were eluted with 0.1 M NaHCO3, 1% SDS at r.t. Immunoprecipitated and input DNA were purified with Proteinase K (Genaxxon) and QIAGEN PCR purification kit.

For Nimblegen Arrays (H3K27ac), 10ng of input (MNase digested) or ChIP DNA were amplified using the WGA2 whole genome amplification kit (Sigma). Amplified material was labeled with Cy3 or Cy5 by random priming according to the NimbleGen ChIP-chip protocol (Roche). Samples were hybridized for 20 h and washed according to manufacturer's protocol. A custom 3x720K mouse tiling array (NimbleGen, Roche) containing 179,493 unique probes from different genomic regions, with each probe represented by 4 replicates was used. Arrays were scanned on a NimbleGen MS 200 Microarray scanner (Roche) using 100% laser power and 2 μm resolution.

ChIP-seq data for H3K27me3 in mESCs were taken from (Illingworth et al., 2015) GEO: GSE69955.

5C primer, 5C library design and preparation

3C library preparation was performed as previously described (Williamson et al., 2014). 1 × 10^7 ESCs or NPCs were fixed with 1% formaldehyde for 10 min at r.t. Cross-linking was stopped with 125 mM glycine for 5 min at r.t. followed by 15 min on ice. Cells were centrifuged at 400 *g* for 10 min at 4°C, supernatants were removed, and cell pellets were flash-frozen on dry ice. Cell pellets were then treated as previously described (Williamson et al., 2016).

Briefly, 1-2 × 10^7 fixed cells were incubated for 15 min on ice in 200 μL of lysis buffer (10 mM Tris pH 8.0, 10 mM NaCl, 0.2% NP40, supplemented with fresh protease inhibitor cocktail). Cells were then disrupted on ice with a Dounce homogenizer (pestle B; 2 × 20 strokes) and cell suspensions centrifuged at 2000 *g* for 5 min. Supernatants were removed, the cell pellets were washed twice with 100 μL of 1 × CutSmart buffer (New England Biolabs), and resuspended in 100 μL of 1 × CutSmart buffer and divided into two Eppendorf tubes. 1 × CutSmart buffer (337 μL) was added to each tube, and the mixture was incubated for 10 min at 65°C with 0.1% SDS. Forty-four microliters of 10% Triton X-100 were added before overnight digestion with 400 U of HindIII. The restriction enzyme was inactivated by adding 86 μL 10% SDS and incubation for 30 min at 65°C. Samples were then individually diluted into 7.62 mL of ligation mix (750 μL 10% Triton X-100, 750 μL 10 × ligation buffer, 80 μL 10 mg/ml of BSA, 80 μL 100 mM ATP, 3000 cohesive end units of T4 DNA ligase) and incubated for 2 h at 16°C.

3C libraries were incubated overnight at 65°C with 50 μL of Proteinase K (10 mg/ml) and an additional 50 μL of Proteinase K the following day for 2 h. The DNA was purified by one phenol and one phenol–chloroform extraction and precipitated with 0.1 vol (800 μl) of 3 M NaOAc (pH 5.2) and 2.5 vol of cold EtOH (20 ml). After at least 1 h at −80°C, the DNA was centrifuged at 20,000 *g* for 25 min at 4°C, and the pellets washed with cold 70% EtOH. DNA was resuspended in 400 μL of TE (pH 8.0) and transferred to Eppendorf tubes for another phenol–chloroform extraction and precipitation with 40 μL of 3 M NaOAc (pH 5.2) and 1.1 mL of cold EtOH. DNA was recovered by centrifugation and washed eight times with cold 70% EtOH. Pellets were then dissolved in 100 μL of TE pH 8.0 and incubated with1 μL of 10 mg/ml RNase A for 15 min at 37°C.

For PARP1 experiments a modified 3C library protocol for low cell numbers (~2 million) was used as described (Berlivet et al., 2013). Essentially this is the same as the standard protocol, the modifications being mainly in the buffer volumes for DNA ligation and purification and the number of alcohol washes.

5C primers covering the USP22 (mm9, chr11: 60,917,307–61,017,307) and Shh (mm9, chr5: 28317087-30005000) regions, library design, and preparation, were performed as described (Williamson et al., 2016). 5C libraries were prepared and amplified with the A-key and P1-key primers (Fraser et al., 2012). 3C libraries were first titrated by PCR for quality control (single band, absence of primer dimers, etc.) and to verify that contacts were amplified at frequencies similar to that usually obtained from comparable libraries. We used 1–11 μg of 3C library per 5C ligation reaction.

5C primer stocks (20 μM) were diluted individually in water on ice and mixed to a final concentration of 0.002 μM. Mixed diluted primers (1.7 μl) were combined with 1 μL of annealing buffer (10 × NEBuffer 4, New England Biolabs) on ice in reaction tubes. Salmon testis DNA (1.5 μg) was added to each tube, followed by the 3C libraries and water to a final volume of 10 μl. Samples were denatured for 5 min at 95°C and annealed for 16 h at 48°C. Ligation with 10 U of Taq DNA ligase was performed for 1 h at 48°C. One-tenth (3 μl) of each ligation was then PCR-amplified individually with primers against the A-key and P1-key primer tails. We used 26 or 28 cycles based on dilution series showing linear PCR amplification within that cycle range. The products from two to four PCR reactions were pooled before purifying the DNA on MinElute columns (QIAGEN).

5C libraries were quantified on agarose gels and diluted to 0.0534 ng/μL (for Xpress template kit version 2.0) or 12 pmol (for Ion Proton). One microliter of diluted 5C library was used for sequencing with an Ion Proton sequencer. Samples were sequenced as recommended by the manufacturer (Life Technologies).

4SU-seq

4SUseq was performed essentially as described previously (Rabani et al., 2011). Briefly, 4-thiouridine (4SU; Sigma, #T4509) was added to ESCs/NPCs in culture to a final concentration of 500 μM and incubated at 37°C for 20 min. Cells were harvested by trypsinisation and washed twice with PBS at r.t. Total RNA was isolated from 5x10^6 cells using Trizol according to the manufacturer's instructions (Invitrogen, #15596026). Following precipitation, purified RNA was resuspended in 100 μL RNase-free water and DNase

treated using the TURBO DNA-free kit according to the manufacturer's instructions (Invitrogen, #AM1907M). Residual inactivation beads were removed by spinning the RNA sample through a QIAshredder column at 1000 *g* for 1 min (QIAGEN, #79654). Following quantification, 50 μg of RNA were incubated for 1.5 h at r.t. with 100 μg of Biotin-HPDP (Pierce, #21341; reconstituted in dimethylformamide at 1 mg/ml) in 1x Biotinylation Buffer (10 mM Tris pH 7.4 and 1 mM EDTA) to a total volume of 500 μl. Uncoupled biotin was removed through two consecutive rounds of 1:1 v/v chloroform extraction followed by isopropanol/NaCl precipitation. RNA was resuspended in 100 μL of RNase free water and mixed 1:1 w/w with μMacs Streptavidin beads (Miltenyi, #130-074-101) and incubated for 15 min at RT with rotation. The RNA / bead mixture was applied to a μMacs column following pre-equilibration with wash buffer (100 mM Tris pH 7.5, 10 mM EDTA, 1 M NaCl and 0.1% Tween20). The captured beads were then washed with 3 × 900 μl of 65°C wash buffer and 3 × 900 μl RT wash buffer. RNA was then eluted from the column by adding two consecutive rounds of 100 mM DTT. The eluate was added to 700 μl Buffer RLT (RNeasy MinElute Cleanup Kit; QIAGEN, #74204) and then purified according to the manufacturer's instructions. Prior to library preparation, ribosomal RNA was depleted from the purified 4SU incorporate RNA using the low Input RiboMinus Eukaryote System v2 kit according to the manufacturer's instructions (Ambion, # A15027).

Sequencing libraries were constructed using the NEBNext® Ultra II Directional RNA Library Prep Kit for Illumina according to the protocol for ribosome depleted RNA and with a 10 min RNA fragmentation step (NEB, #E7760). Library PCRs were supplemented with 2x SYBR dye (Sigma, #S9430) so that amplification could be monitored by quantitative PCR on a Roche lightcycler 480. To allow for sample multiplexing, PCRs were performed using index primers (NEBNext Multiplex Oligos for Illumina - Set 1. #E7335) and amplified to linear phase. Libraries were purified and then combined into 4 sample equimolar pools containing the indexes 1, 3, 6 and 8.

QUANTIFICATION AND STATISTICAL ANALYSIS

FISH image analysis

SIM image processing and reconstruction were carried out using the N-SIM module of NIS-Element Advanced Research software. Images were reconstructed using NiS Elements software (Nikon Instruments) from a *z*-stack comprising of no less than 1 μm of optical sections. In all SIM image reconstructions the Wiener and Apodization filter parameters were kept constant.

Image analysis was carried out using the Quantitation module of Volocity (PerkinElmer). Reconstructed SIM data was directly uploaded and analyzed on Volocity. The statistical significance of differences in inter-probe distances was assessed using the nonparametric Mann-Whitney U test to examine the null hypothesis. Bimodality analysis was done using Hartigan's dip test. Each dataset consisted of 20 to 50 nuclei (40 to 100 loci). Biological replicates are shown under their p values in Supplemental figures and Tables.

qRT-PCR

The relative expression of each sample was measured by the Lightcycler software and normalized to the mean for *Gapdh* from replicates. The log2 of the ratio relative to eGFP transfected ESCs was calculated.

For single-cell qRT-PCR, 3 plates were used for technical replicates. Data analysis was performed as for regular qRT-PCR using the first well (32 cells) of ESCs and NPCs plates as a mean for *Gapdh* and *Shh*. For data analysis, wells without data for *Gapdh* or *Shh* were removed. Values normalized to *Gapdh* were then plotted in to R using a density plot.

Microarray analysis

Raw signal intensities were quantified from TIFF images using MS 200 Data Collection software. ChIP microarray data were analyzed in R using the bioconductor package Limma. Raw signal intensity values were averaged across replicate probes (4 replicated per unique probe sequence). Averaged signal intensities were subsequently corrected for dye bias between each pair of co-hybridized ChIP and input samples using loess normalization. Corrected values were then transformed into log2 ChIP/Input ratios and scale normalized. For visualization, normalized log2 intensity ratios were assigned to the mm9 location of the probe sequence.

5C analysis

Analysis of 5C sequencing data was performed as described previously (Berlivet et al., 2013). Sequencing data were processed through a Torrent 5C data transformation pipeline on Galaxy (https://usegalaxy.org). NPC-enriched and TALE activated-enriched 5C interactions were obtained by subtracting wild-type ESC 5C-seq data (Williamson et al., 2014). Before normalizing, interactions between adjacent fragments were removed due to the high noise: signal ratio likely to occur here. Data were normalized by dividing the number of reads of each 5C contact by the total number of reads from the corresponding sequence run. All scales shown correspond to this ratio multiplied by 10^3. Sequencing technical and biological replicates reads are displayed in Table S10.

4SU-seq analysis

Pooled 4SU libraries were sequenced on an Ilumina NextSeq 550 and the resulting fastq files were aligned to the mouse genome (mm9) using bowtie2 v2.2.6 with standard parameters for paired end sequence data to generate .SAM files. Mapped data was processed using HOMER v4.8. SAM files were converted into tag directories using 'makeTagDirectory' with the following parameters: -format sam -flip –sspe. Genomic intervals which extended beyond the end of the chromosomes were

removed using 'removeOutOfBoundsReads.pl'. Strand specific browser track files (.bigWig) were generated by combining replicate tag directories with the 'makeTagDirectory' function and then running 'makeUCSCfile' with the following parameters: -fsize 1e20 -strand + (or -) -norm 1e8 -color 25,50,200.

DATA AND CODE AVAILABILITY

Data from this paper are available at NCBI GEO under the series GSE89557 which includes sub-series for the 5C (GSE89388), ChIP-chip (GSE115642) and 4SU-seq data (GSE115774).

Molecular Cell, Volume *76*

Supplemental Information

Decreased Enhancer-Promoter Proximity Accompanying Enhancer Activation

Nezha S. Benabdallah, Iain Williamson, Robert S. Illingworth, Lauren Kane, Shelagh Boyle, Dipta Sengupta, Graeme R. Grimes, Pierre Therizols, and Wendy A. Bickmore

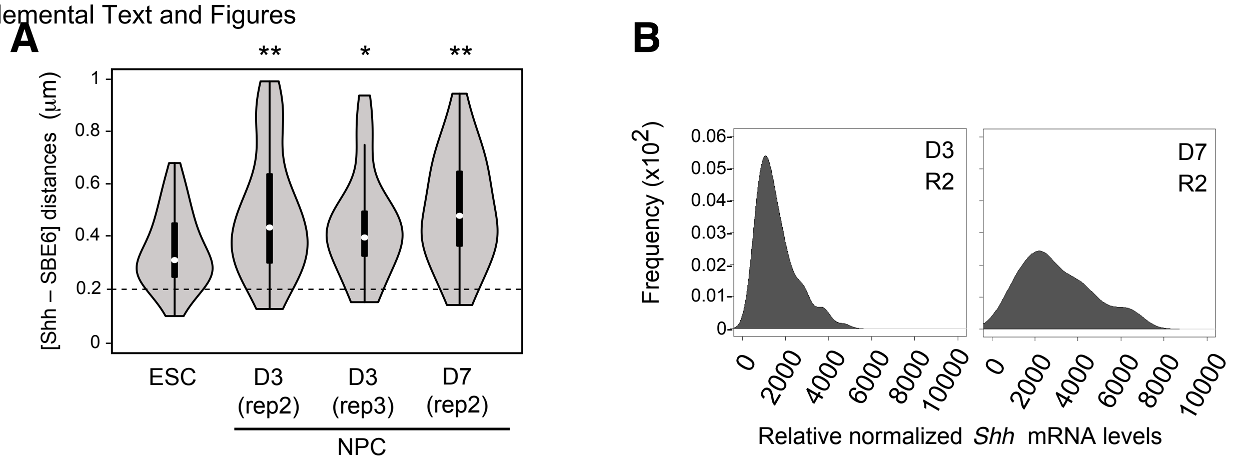

Supplementary Figure 1

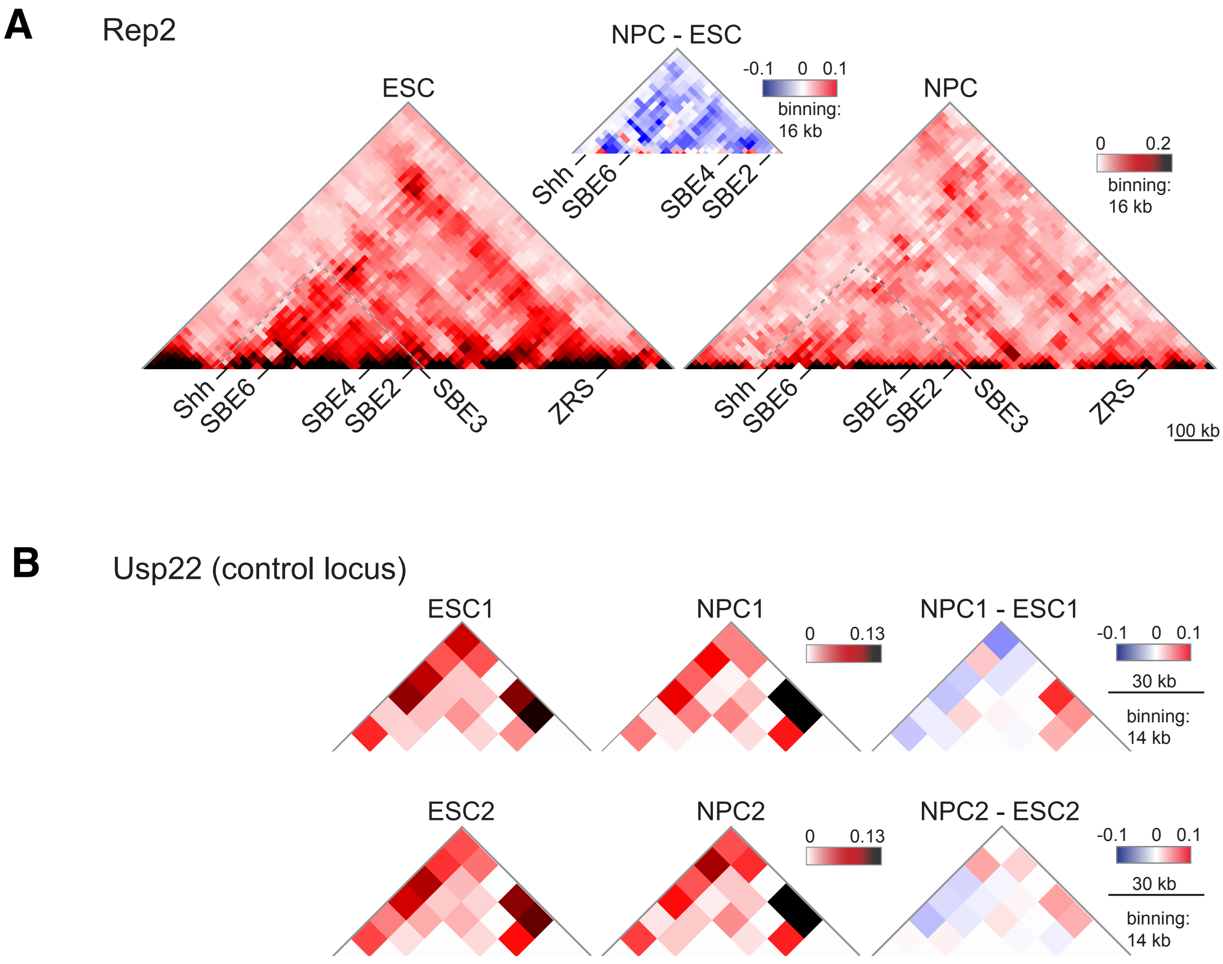
A
Rep2
NPC - ESC
-0.1 0 0.1
binning: 16 kb
ESC
NPC
0 0.2
binning: 16 kb
Shh
SBE6
SBE4
SBE2
SBE3
ZRS
100 kb
B
Usp22 (control locus)
ESC1
NPC1
NPC1 - ESC1
0 0.13
-0.1 0 0.1
30 kb
binning: 14 kb
ESC2
NPC2
NPC2 - ESC2

Figure S2

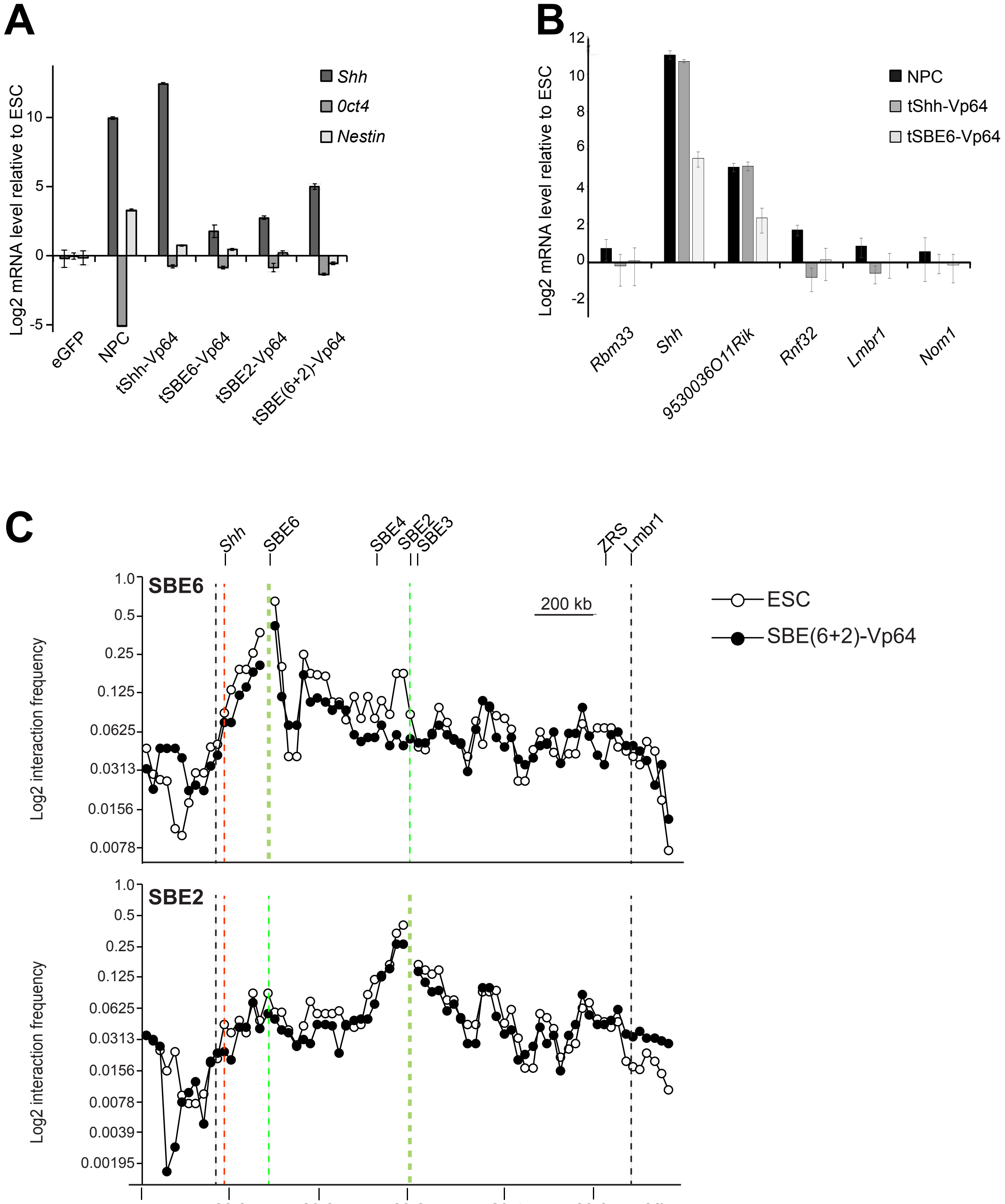
A
Log2 mRNA level relative to ESC
Shh
0ct4
Nestin
eGFP
NPC
tShh-Vp64
tSBE6-Vp64
tSBE2-Vp64
tSBE(6+2)-Vp64
B
NPC
tShh-Vp64
tSBE6-Vp64
Rbm33
Shh
9530036O11Rik
Rnf32
Lmbr1
Nom1
C
Shh
SBE6
SBE4
SBE2
SBE3
ZRS
Lmbr1
SBE6
200 kb
ESC
SBE(6+2)-Vp64
Log2 interaction frequency
SBE2
28.8
29.0
29.2
29.4
29.6
Mb

Figure S3

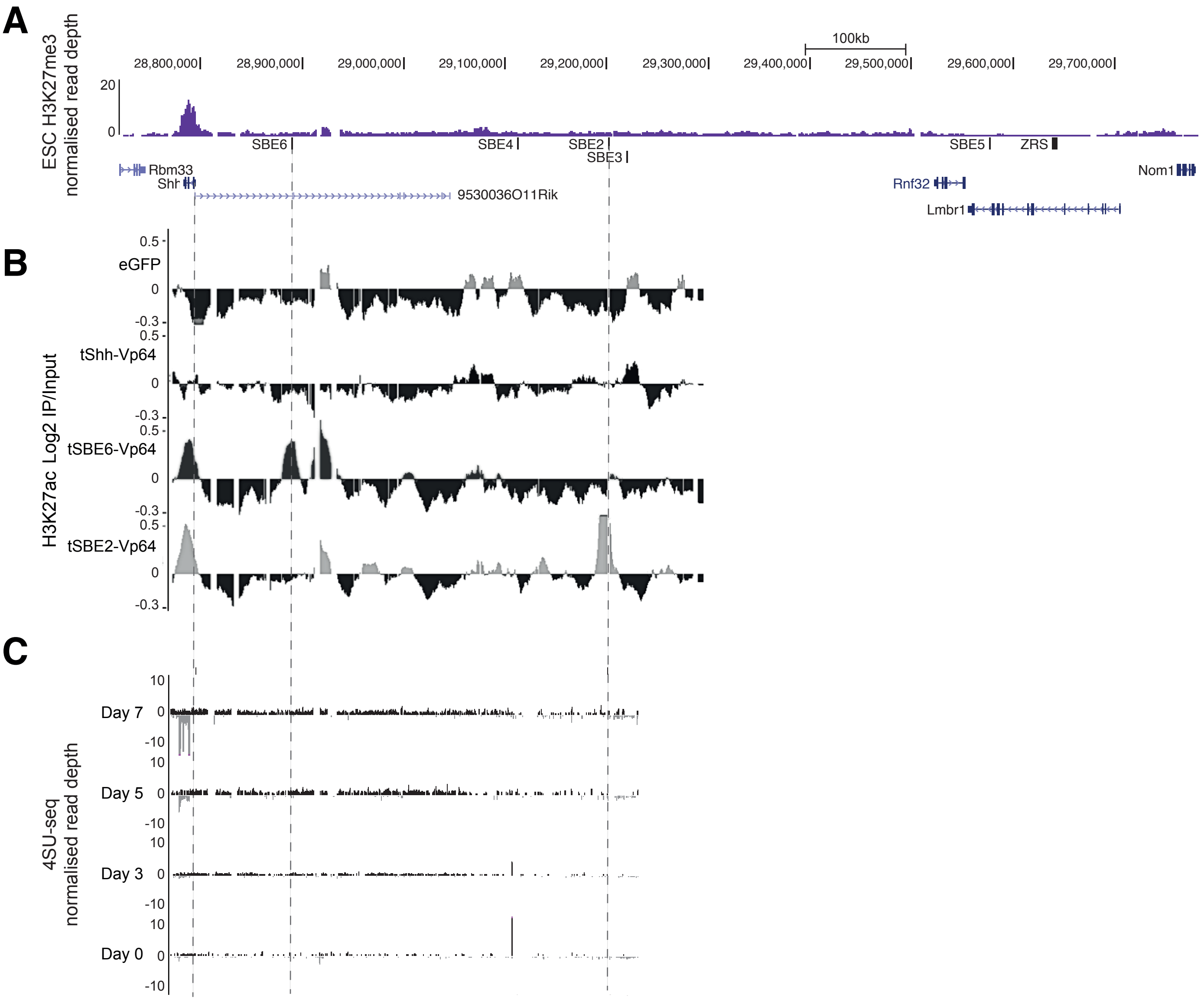

Supplementary Figure 4

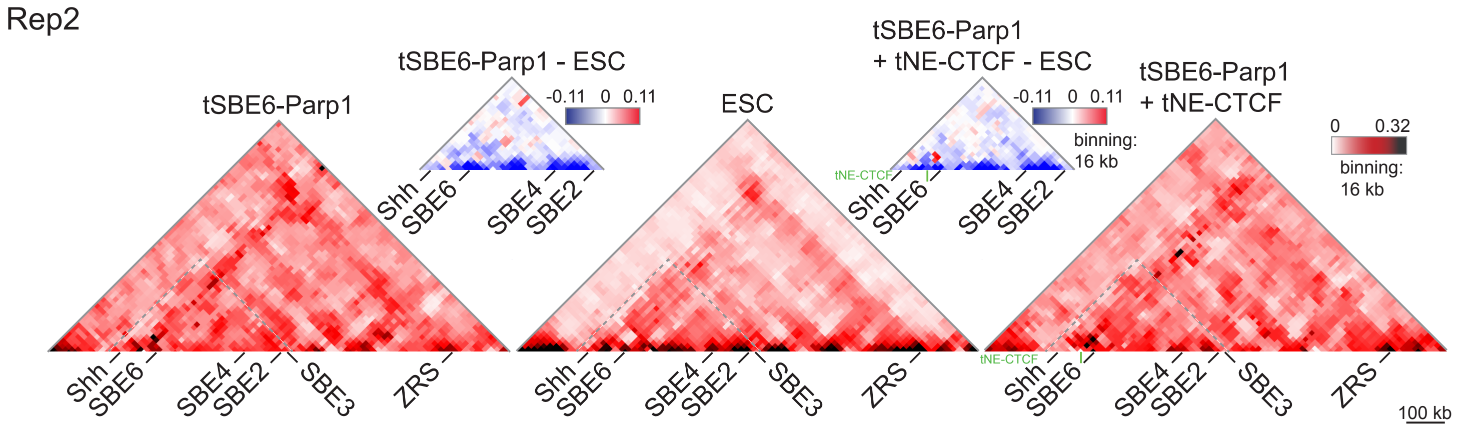
Rep2
tSBE6-Parp1
tSBE6-Parp1 - ESC
-0.11 0 0.11
ESC
tSBE6-Parp1
+ tNE-CTCF - ESC
-0.11 0 0.11
binning:
16 kb
tSBE6-Parp1
+ tNE-CTCF
0 0.32
binning:
16 kb
Shh
SBE6
SBE4
SBE2
SBE3
ZRS
tNE-CTCF
100 kb

Figure S5

Supplementary Figures

Figure S1. FISH and Shh expression during NPC differentiation. Related to Figure 1.

A) Violin plots representing Shh-SBE6 inter-probe distances after FISH in ESCs and in NPCs differentiated for 3 (D3) and 7 (D7) days of differentiation. Data are from the second (rep 2) and third (rep 3) biological replicates of the experiment shown in Figure 1. * $p < 0.05$, ** $p < 0.01$. B) Kernel density plots showing *Shh* mRNA expression in single NPCs relative to *Gapdh* and normalised to expression in ESC at D3 and D7 of differentiation for a second biological replicate. Statistical data relating to FISH data from this figure are included in Table S1.

Figure S2. Chromosome conformation capture of the Shh regulatory region. Related to Figure 2.

A) 5C heat-maps from ESCs and NPCs for the *Shh* regulatory region (mm9, chr5:28604000-29780000) with 16kb binning and smoothing (biological replicate of experiment in Figure 2). Difference 5C plots are shown above the main 5C plots. B) 5C heat maps from the control Usp22 locus.

Figure S3. Inducing Shh expression in ESCs does not perturb pluripotency. Related to Figure 3.

A) qRT-PCR showing mean (± SEM) log2 mRNA levels of *Shh*, *Oct4* and *Nestin* relative to *Gapdh* and normalized to eGFP expressing ESCs, in NPCs and in ESCs expressing tShh-Vp64, tSBE6-Vp64, tSBE2-Vp64 and tSBE(6+2)-Vp64. Data are from one biological replicate. B) As in (A) but for genes from the Shh TAD and the flanking TADs. Data in A and B relate to main Figure 3. C) Virtual 4C visualisation of 5C data from main Figure 3E, for ESCs and for ESCs expression SBE(6+2)-Vp64 with SBE6 and SBE2 viewpoints. Genome co-ordinates (Mb) are from the mm9 assembly of the mouse genome.

Figure S4. Chromatin and transcription profiling of the Shh regulatory region. Related to Figure 3.

A) Normalised read counts for H3K27me3 ChIP-seq across the *Shh* regulatory region. The position of Shh enhancers (SBE2-6 and ZRS) as well as the position of genes in the region is shown below the ChIP track. Genome co-ordinates are from the mm9 assembly of the mouse genome. NCBI GEO accession number for these data: GSE89512. Data are from Illingworth et al., 2015; GEO accession GSE69955). B) Log2 ratio H3K27ac ChIP: input of MNase digested chromatin, hybridised to an array covering the

Shh regulatory region. Mapping is aligned to the genome browser track shown in (A). Data are from; ESCs expressing control eGFP, or TALE-Vp64 fusions targeting the *Shh* promoter (tShh-Vp64), SBE6 (tSBE6-Vp64) or SBE2 (tSBE2-Vp64). Dotted lines represent location of the *Shh* promoter, SBE6 and SBE2. NCBI GEO accession number for these data: GSE89512. C) Normalised read counts for 4SU-seq during a time course of NPC differentiation from day 0 to day 7. Tracks are aligned to the genome co-ordinates in (A). NCBI GEO accession number for these data: GSE115774.

Figure S5. Chromosome conformation capture of the* Shh *regulatory region in wild type, tSBE6-PARP1 and tSBE6-PARP1 + tNE-CTCF ESCs. Related to Figure 6.

Biological replicate for experiment in Figure 6G. 5C heatmaps of the *Shh* regulatory region (mm9, chr5: 28604000-29780000) with 16kb binning and smoothing. Difference 5C plots are shown above the main 5C plots.

Table S1, Mann-Whitney U significance tests for inter-probe FISH distances across the Shh regulatory domain during NPC differentiation. Related to Figure 1

Table showing the *p*-values of each biological replicate for differences between the inter-probe distances from ESCs against the data from various days of NPC differentiation. Values in bold are significant ($p<0.05$). Also shown are the % alleles (probe pairs) where probes are considered co-localised (i.e. distances of <200nm) for data from ESCs and NPCs. 40 to 100 loci were analysed per experiment. Unless otherwise indicated, imaged were captured using structured illumination microscopy (SIM).

**Probe pairs**	**Day of NPC differentiation**	***p***	**Imaging mode**	**% of probe pair <200nm in ESCs**	**% of probe pair <200nm in NPCs**
**Shh-SBE6**	**3**	0.55	SIM	26.3	25.4
		**0.018**	SIM	11.5	7.5
		**0.002**	SIM	11.5	9.7
**Shh-SBE6**	**4**	**0.011**	SIM	26.3	18.1
**Shh-SBE6**	**5**	**<0.001**	SIM	26.3	13.5
**Shh-SBE6**	**6**	**<0.001**	SIM	26.3	11.0
	**7**	**0.033**	SIM		
		**0.020**	SIM		
		**0.003**	SIM		
		**0.002**	SIM		
**Shh-SBE6**		**<0.001**	SIM	26.3	19,2
		**<0.001**	SIM	11.5	4.3
		**<0.001**	SIM	25.3	9.1
		**<0.001**	SIM	7.8	3.8
		**<0.001**	SIM	15.8	3.9
		**<0.001**	SIM	26	3.6
		**<0.001**	SIM	13.6	4.9
**Shh-SBE4**	**7**	**0.018**	Widefield		
		**0.015**	Widefield		
		**<0.001**	SIM	23.8	6.6
		**<0.001**	SIM	10.6	5.4
**Shh-SBE2/3**	**7**	0.946	SIM		
		0.644	SIM	17.5	18
		0.529	SIM		
		0.493	SIM		
**Shh-ZRS**	**7**	0.057	Widefield		
		0.656	SIM		
		0.594	SIM		
		0.388	SIM		
**Shh-Control**	**7**	0.680	Widefield	Widefield	Widefield
		0.432	SIM		

Table S2. Mann-Whitney U significance tests for Shh-*SBE6 *inter-probe distances between eGFP and TALE-Vp64/TALE-Vp128 transfected cells. Related to Figure 3.

p-values of each biological replicate of the data from Figures 3C and D are shown for the differences between the inter-probe distances from ESCs expressing a control eGFP construct and the data from ESCs expressing TALE-Vp64/Vp128 constructs. Values in bold are significant ($p<0.05$). Also shown are the % alleles (probe pairs) where probes are considered co-localised (i.e. distances of <200nm).

TALE-Vp64/Vp128 constructs	Shh-SBE6 *p* value	% of probe pair <200nm in eGFP control	% probe pairs <200nm in TALE-fusions
**tShh-Vp128**	0.440	26.6	35.8
	0.073	31.5	8.9
**tSBE6-Vp128**	**0.001**	31.5	**7.5**
**tSBE2-Vp128**	**0.0021**	**22.2**	**1.8**
	**<0.001**	31.5	**0**
**tSBE(6+2)-Vp64**	**0.045**	**8.4**	**5.6**
	**0.013**	**28.3**	**9.6**
	**<0.001**	**32.9**	**3.6**
	**<0.001**	**25.7**	**2.9**
	**<0.001**	**29.5**	**3.5**
	**<0.001**	**31.1**	**18.4**
	**<0.001**	**25.7**	**2.9**
	**<0.001**	**30.8**	**4.1**
**tSBE(6+2)-Δ**	0.228	28.3	28.3

Table S3, Mann-Whitney U significance tests for Shh-*SBE6 *inter-probe distances across the Shh regulatory domain after expression of TALE-LDB1. Related to Figure 3.

Table showing the *p*-values of each biological replicate for differences between the inter-probe distances between the data from ESCs expressing a control eGFP construct and the data from ESCs expressing TALE-LDB1 constructs. Data are from Figures 3H and I. Values in bold are significant ($p<0.05$). Also shown are the % of alleles (probe pairs) where probes are considered as co-localised (i.e. distances of <200nm) for data from ESCs and cells expressing the various TALE-LBD1 fusions.

Probe pairs	TALE-LDB1 constructs	*p* value	% of probe pair <200nm in ESCs	% of probe pair <200nm in TALE-LBD1
**Shh-SBE6**	**tShh-LDB1 + tSBE6-LDB1**	**0.022**	36.3	54.4
	**tShh-LDB1 + tSBE2-LDB1**	0.457	17.7	21.2
		0.167	22.6	17.2
**Shh-SBE2/3**	**tShh-LDB1 + tSBE2-LDB1**	**0.017**	22.1	34.8
		**<0.001**	18.6	45.5
		**<0.001**	10.5	35.1
**SBE6-SBE2/3**	**tShh-LDB1+tSBE2-LDB1**	0.895	29.1	19.4
		0.198	28.8	29.5
	**tSBE6-LDB1+tSBE2-LDB1**	**0.018**	23.7	48.8

Table S4, Mann-Whitney U significance tests for Shh-SBE6 inter-probe distances after expression of TALE-VWA or SIX3. Related to Figure 4.

p-values of each biological replicate of the data from Figure 4 are shown for the differences between the inter-probe distances from ESCs expressing a control eGFP construct and those from ESCs expressing TALE-VWA or Six3 constructs. Values in bold are significant (p<0.05). Also shown are the % alleles (probe pairs) where probes are considered co-localised (i.e. distances of <200nm).

**TALE-constructs**	**Shh-SBE6 *p* value**	**% probe pairs <200nm in eGFP control**	**% probe pairs <200nm in TALE-fusions**
tShh-VWA	0.359	17.4	20.4
tSBE(6+2)-VWA	**0.04**	**17.4**	**4.3**
	**<0.001**	**18.6**	**2.6**
tShh-SIX3	0.594	22.6	29.6
tSBE2-SIX3	**0.012**	**22.6**	**21.4**
	**<0.001**	**20.6**	**8.3**
	**<0.001**	**33.3**	**9.1**

Table S5, Mann-Whitney U significance tests for Shh-SBE6 inter-probe distances after expression of TALEs-that target NE. Related to Figure 5.

p-values of each biological replicate are shown for the differences between the Shh-SBE6 inter-probe distances from ESCs expressing a control eGFP construct and from ESCs expressing various TALE-constructs. Data from Figure 5.

**TALE-constructs**	**Shh-SBE6 *p* value**
tSBE(6+2)-Vp64 + tNE-CTCF	0.626<br>0.602
tSBE(6+2)-Vp64 + tNE-Δ	0.596<br>0.168
tSBE(6+2)-VWA + tNE-Δ	0.627

Table S6. Mann-Whitney U significance tests for Shh-SBE6 inter-probe distances after TALE-fusion expression and after treatment with olaparib. Related to Figure 6.

p-values of each biological replicate are shown for differences between the inter-probe distances from ESCs expressing a control eGFP construct against the data from ESCs expressing TALE constructs, or from NPCs treated with olaparib. Data relate to Figure 6C-F. Values in bold are significant ($p<0.05$).

TALE-fusion constructs	Shh-SBE6 *p* value
tSBE6-PARP1	**0.001**
tSBE6-PARP1 + tNE-CTCF	0.106
tSBE2-PARP1	**<0.001**<br>**<0.001**
tSBE2-PARP1 + tNE-CTCF+	0.883
tSBE2-SIX3	**0.012**<br>**<0.001**<br>**<0.001**
tSBE2-SIX3 + Olaparib	0.997<br>0.629<br>0.204
tSBE2-PARP1+Olaparib	0.816<br>0.680<br>0.417
NPC (d7) + Olaparib	0.799<br>0.546
tShh-DEL	**0.003**
tSBE2-DEL	**<0.001**<br>**<0.001**
tSBE2-DEL + Olaparib	**<0.001**<br>**<0.001**

Table S7. Mann-Whitney U significance tests for Shh-SBE6 inter-probe FISH distances across the Shh regulatory domain after recruitment of wild-type and mutant PARP1. Related to Figure 6.

p-values for the differences between the Shh-SBE6 inter-probe distances from ESCs expressing a control eGFP construct and from ESCs expressing various TALE-PARP1 fusion constructs, either wild-type PARP1, or catalytically inactive mutant PARP1s (E988K or D890K+M899N). Data relate to Figure 6H. Values in bold are significant ($p<0.05$).

Probe pairs	TALE-fusion constructs	*p* value
**Shh-SBE6**	tSBE6-PARP1	**<0.001**
	tSBE2-PARP1	**<0.001**
	tSBE6-E988K	0.168<br>0.337
	tSBE6-D890V+M899N	0.794<br>0.226
	tSBE2-E988K	0.756<br>0.447
	tSBE2-D890V+M899N	0.984<br>0.7414

Table S8: Enhancer and FISH probe co-ordinates. Related to STAR Methods section 3D-FISH

The table lists the genome co-ordinates (bp) on chromosome 5 (mm9 assembly of the mouse genome) of *Shh* and its enhancers, and the fosmid probes used for FISH.

Locus	Gene/ enhancer	Fosmid probes			
		start (bp)	end (bp)	fosmid ID	Clone name
CTRL		28411516	28449628	G135P60453C9	WI1-2806K8
Shh	28783380-28793641	28754458	28795879	G135P64333A4	WI1-574O18
SBE6	28889688-28890461	28887686	28924744	G135P67311F4	WI1-442E17
SBE4	29111752-29112865	29107140	29147593	G135P600205H10	WI1-2751A06
SBE2/3	29202018-29219606	29195832	29239355	G135P603171G8	WI1-1275C09
ZRS	29638200-29643599	29611727	29653695	G135P600929F6	WI1-1047E14

Table S9: Table of PCR primers for qRT-PCR and Chip qPCR. Related to STAR Methods section

**qRT-PCR primers**	
Gapdh Fw	ATCACCATCTTCCAGGAGCGAG
Gapdh Rv	GACCCTTTTGGCTCCACCCTTC
Oct4 (Pou5f1) Fw	CGAGAACAATGAGAACCTTC
Oct4 (Pou5f1) Rv	CCTTCTCTAGCCCAAGCTGAT
Nestin Fw	GATCGCTCAGATCCTGGAAG
Nestin Rv	AGGTGTCTGCAAGCGAGAGT
Shh Fw	ACGATTTAAGGAACTCACCC
Shh Rv	TTGTCTTTGCACCTCTGAG
9530036O11Rik Fw	GCCTGAAACACACAGAATGC
9530036O11Rik RV	GATGGGAGAACTCAGCCAAG
Parg Fw	GAAGAAGTGGCTGGGAACTCCT
Parg Rv	GTTTCGGAACCTCTCCTGCTCT
**ChIP qPCR primers**	
tNE Fw	CAAGAAAACATGGGTCTAGGG
tNE Rv	TGAATTGGAACATTTCTTCAGG
tShh Fw	AAGGCTGGAGAGCTTGTGAG
tShh Rv	TAAAGCCACAGCAGCCAGAG
Actin Fw	CCTCGATGCTGACCCTCATCC
Actin Rv	GACACTGCCCCATTCAATGTCTC

Table S10: 5C sequencing technical and biological replicates reads. Related to STAR Methods section

Experiment	No. of reads	No. of used reads	No. of used reads *Shh* reg. region
ESC biological replicate 1 (tSBE-Vp64 experiments)	45,024,284	23,168,046	13,752,662
ESC biological replicate 1	1,745,058	1,368,704	844,392
ESC biological replicate 2	3,606,454	2,830,120	1,751,368
NPC biological replicate 1-technical replicate 1	3,984,330	1,627,144	795,658
NPC biological replicate 1-technical replicate 2	9,814,008	4,168,546	2,267,024
NPC biological replicate 2-technical replicate 1	2,079,276	894,214	411,396
NPC biological replicate 2-technical replicate 2	92,081,330	33,796,970	15,038,310
NPC biological replicate 2-technical replicate 3	16,481,938	5,648,428	2,705,028
tSBE6-Vp64 biological replicate 1	90,452,930	50,035,502	30,790,810
tSBE2-Vp64 biological replicate 1	1.01E+08	44,074,854	21,016,670
tSBE(6+2)-Vp64 biological replicate 1-technical replicate 2	79,792,180	32,329,736	16,415,488
tSBE6-PARP1 biological replicate 1	2,066,022	1,002,156	562,782
tSBE6-PARP1 biological replicate 2	1,664,928	1,255,928	728,276
tSBE6-PARP1 + tNE-CTCF biological replicate 1	2,478,062	1,873,946	1,173,160
tSBE6-PARP1 + tNE-CTCF biological replicate 2	1,604,560	1,231,786	706,104
